# Efficient Inference of Recombination Hot Regions in Bacterial Genomes

**DOI:** 10.1093/molbev/msu082

**Published:** 2014-02-27

**Authors:** Koji Yahara, Xavier Didelot, M. Azim Ansari, Samuel K. Sheppard, Daniel Falush

**Affiliations:** ^1^Department of Medical Genome Sciences, Graduate School of Frontier Sciences, University of Tokyo, Tokyo, Japan; ^2^Institute of Medical Science, University of Tokyo, Tokyo, Japan; ^3^Institute of Life Science, College of Medicine, Swansea University, Swansea, United Kingdom; ^4^Department of Infectious Disease Epidemiology, Imperial College London, London, United Kingdom; ^5^Department of Statistics, University of Oxford, Oxford, United Kingdom; ^6^Department of Evolutionary Genetics, Max Planck Institute for Evolutionary Anthropology, Leipzig, Germany

**Keywords:** recombination hotspot, recombination hot region, homologous recombination, selection, population genomics, chromosome painting

## Abstract

In eukaryotes, detailed surveys of recombination rates have shown variation at multiple genomic scales and the presence of “hotspots” of highly elevated recombination. In bacteria, studies of recombination rate variation are less developed, in part because there are few analysis methods that take into account the clonal context within which bacterial evolution occurs. Here, we focus in particular on identifying “hot regions” of the genome where DNA is transferred frequently between isolates. We present a computationally efficient algorithm based on the recently developed “chromosome painting” algorithm, which characterizes patterns of haplotype sharing across a genome. We compare the average genome wide painting, which principally reflects clonal descent, with the painting for each site which additionally reflects the specific deviations at the site due to recombination. Using simulated data, we show that hot regions have consistently higher deviations from the genome wide average than normal regions. We applied our approach to previously analyzed *Escherichia coli* genomes and revealed that the new method is highly correlated with the number of recombination events affecting each site inferred by ClonalOrigin, a method that is only applicable to small numbers of genomes. Furthermore, we analyzed recombination hot regions in *Campylobacter jejuni* by using 200 genomes. We identified three recombination hot regions, which are enriched for genes related to membrane proteins. Our approach and its implementation, which is downloadable from https://github.com/bioprojects/orderedPainting, will help to develop a new phase of population genomic studies of recombination in prokaryotes.

## Introduction

Recombination is a fundamental driving force in evolution. Patterns of recombination have been studied most actively in humans, revealing considerable variation of recombination rates across the genome with recombination “hotspots” in which the majority of crossover occurs (McVean et al. 2004; Myers et al. 2005). A high-resolution genetic map of recombination was recently inferred in African Americans, based on individuals who experienced recent genetic admixture together with the unadmixed reference populations (Price et al. 2009; Hinch et al. 2011). A fine-scale map of recombination hotspots was also constructed in chimpanzee (Auton et al. 2012).

Meanwhile, although studies have shown variation in recombination between bacterial species (Smith et al. 1993; Narra and Ochman 2006; Vos and Didelot 2009), between different lineages within the same species (Rocha et al. 2005; Castillo-Ramirez et al. 2012; Joseph et al. 2012; Croucher et al. 2013), knowledge of variation in recombination rate across a bacterial genome is relatively limited. Homologous recombination in bacteria results in the import of short contiguous DNA fragments, which is similar to gene conversion in eukaryotes (Smith et al. 1991; Didelot and Falush 2007a; Takuno et al. 2012). Here, we focus on the problem of identifying recombination hot regions, defined as locations of the genome where DNA is transferred frequently between isolates. This is not the same as locating hotspots, that is, regions of the genome where recombination breakpoints occur frequently. For example, in *Neisseria meningitidis*, preferential recombination of DNA fragments spanning the TbpB (transferrin-binding protein B) gene has previously been reported, presumably due to selection for host immune escape (Linz et al. 2000). Many different recombinational breakpoints were observed for the imported fragments, occurring both up and downstream of the gene, suggesting that the hot region is not due to specific hotspots around the gene but rather to natural selection for different variants of the TbpB gene.

In addition to natural selection, recombination rates can vary along the genome due to specificities of the mechanisms that facilitate DNA transfer. Particular chromosomal regions of the genome might be more susceptible to recombination, for example, due to their positions relative to the origin of replication. Enzymes in the DNA replication and repair machinery (e.g., the RecBCD enzyme [Dillingham and Kowalczykowski 2008]) target specific sequences (e.g., the chi sequence [Anderson and Kowalczykowski 1997; Handa et al. 2012] and specific DNA uptake sequence [Treangen et al. 2008; Frye et al. 2013]), and this can lead to them being transferred more or less often in recombination events.

The increased availability of sequenced genomes creates an opportunity to conduct data-driven inference of variation in recombination rate across a bacterial genome. However, the methods for eukaryotes are focused on finding hotspots (Auton and McVean 2007; McVean and Auton 2007), and there have been less methodological studies in statistical genomics for the gene conversion-like recombination in bacteria compared with those for crossing over in eukaryotes. A pioneering method explicitly modeling bacterial recombination is ClonalFrame (Didelot and Falush 2007a), which has been successfully used to reveal recombination events across genomes of various bacterial species (Didelot et al. 2011; Joseph et al. 2012; Namouchi et al. 2012; Sheppard, Didelot et al. 2012). However, ClonalFrame does not model the origin of DNA imported in homologous recombination events, which means that it misses many events and is also likely to be inaccurate in inferring recombination boundaries. A related statistical genetic method, ClonalOrigin, directly models bacterial recombination as an event from a specific donor to a specific recipient, so that it simultaneously detects events and their origins (Didelot et al. 2010). ClonalOrigin was recently applied to *Escherichia coli* genomes, which revealed detailed flux of recombination between donors and recipients throughout the genome, and three recombination hot regions in which deviations from clonal descent were significantly more frequent than elsewhere in the genome (Didelot, Méric, et al. 2012).

There are two major difficulties with these methods. First, ClonalOrigin requires a clonal genealogy to be specified, which can be accurately inferred by ClonalFrame only if the recombination rate is not too high (Didelot and Falush 2007b). Second, both methods are computationally expensive and therefore not applicable to hundreds or thousands of bacterial genomes (Loman et al. 2012). Although ClonalFrame is applicable to around 100 genomes if they are not too genetically diverse (Didelot, Eyre, et al. 2012), the computation takes at least several days.

In this study, we present a new approach that overcomes some of the difficulties associated with existing methods for investigating recombination in bacteria. Our approach is based on the recently developed “chromosome painting” algorithm. Under the approach, a hidden Markov model (HMM) is used to “paint” a “recipient” haplotype as a series of chunks from a panel of “donor” haplotypes from other individuals in the sample based on sequence similarity between donor and recipient. The interpretation of the painting is that the donor at a given region of the genome has the most recent shared common ancestor with the recipient individual among all of the possible donors in the panel. Changes in the identity of the donor along the sequence reflect recombination events that lead to different genealogical histories for different parts of the genome. (Lawson et al. 2012). The algorithm can be run separately for different recipient individuals, making it applicable to hundreds or thousands of genomes via parallelization. Lawson et al. used the algorithm to summarize information of genome-wide single-nucleotide polymorphisms (SNPs) into chunks based on a “co-ancestry matrix,” which tabulated the number of chunks from each donor to each recipient individual. The data reduction from a haplotype matrix to a coancestry matrix enables model-based clustering using fineSTRUCTURE (Lawson et al. 2012). The two-step approach was recently demonstrated to be effective not only in human but also in the highly recombining bacterium *Helicobacter pylori* (Yahara et al. 2013).

The chromosome painting algorithm in the form used by Lawson et al. (2012) was intended for identifying recent shared ancestry within a freely recombining population. Each individual was painted using all of the other individuals in the sample as donors. In the context of a more clonal organism, this implementation discards a large amount of information in the presence of close relatives. Because each individual is painted using all of the others as donors, the clonally related recipient isolates will be inferred to receive almost all of their genome from their closest relatives. Recombination events that distinguish them may be identified, but all recombination events in the history of their common ancestor will be obscured.

**Fig. 1.**
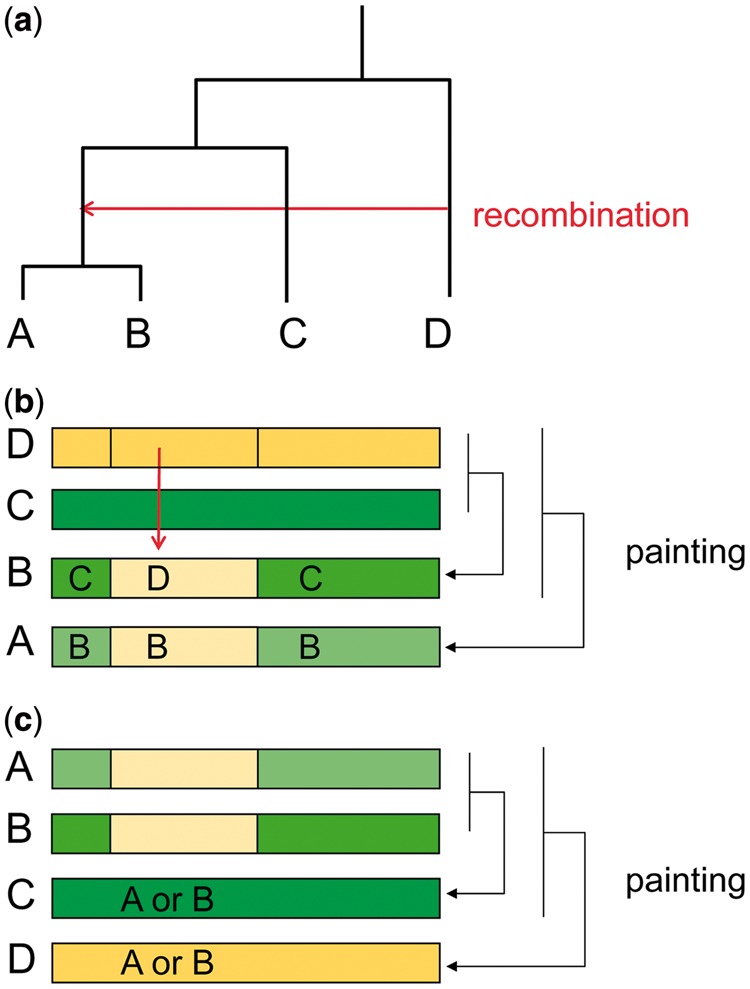
A conceptual example of the ordered painting. (*a*) A recombination event from D to the common ancestor of A and B. (*b*) In the case of ordering D, C, B, A. The red arrow indicates the recombination event from D to B inferred in this ordering. (*c*) In the case of ordering A, B, C, D.

To extract more information from the data than provided by the all-versus-all painting, we generate a random ordering and paint each individual using only individuals preceding it in the ordering (Li and Stephens 2003). This means that if two genomes are closely related, one of the pair will be painted without using the other. This strategy gives different results, depending upon the choice of ordering. For example, suppose that there is a recombination event at one part of the genome from a bacteria closely related to D to the common ancestor of A and B but not their more distant relative C (fig. 1*a*). If the ordering is D, C, B, A, then there will be a donor switch from C to D in the ancestry of B, although there will be no donor switch in the ancestry of A which will copy from B throughout the sequence (fig. 1*b*). If the ordering is A, B, D, C, no recombination event will be detected (fig. 1*c*). We therefore generate multiple orderings for each data set and average over them appropriately.

Here, we illustrate the use of the ordered painting method to infer recombination hot regions in three different data sets: 1) simulated data of a closed recombining population; 2) whole genomes of 27 *E. coli* isolates, which were recently analyzed by ClonalOrigin; and 3) genomes of 200 *Campylobacter jejuni* isolates. The program to perform the inference is called “orderedPainting” and is publicly available from https://github.com/bioprojects/orderedPainting (last accessed March 7, 2014).

## New Approaches

### Ordered Painting

The chromosome painting algorithm is based on the HMM introduced by Li and Stephens (2003). It regards a single haplotype on the chromosome of a recipient individual as a mosaic and reconstructs it as a series of chunks in the sample of potential donors. A “chunk” refers to a set of contiguous SNP(s) copied from a donor to a recipient, bounded by recombination sites beginning of another chunk from a different donor. The donor of each chunk represents a nearest neighbor of the recipient haplotype for that stretch, with each chunk representing a different nearest neighbor. In Lawson et al., the chromosome painting algorithm used an approach in which a recipient haplotype is reconstructed using the haplotypes from all other individuals as potential donors (i.e., “all-versus-all” painting). The reconstruction (painting) process is repeated for every recipient haplotype. For that purpose, donors of each SNP are modeled as hidden states of the HMM, in which transition probabilities depend on recombination rate and distance between SNPs, and transmission probabilities depend on a per site mutation rate. The mutation parameter is fixed as Li and Stephens (2003), and the recombination parameter is inferred from data. A detailed mathematical formulation of the chromosome painting algorithm is given in supporting information of Lawson et al., which applies not only to the all-versus-all painting but also to the conditioning below.

Here, we conduct the chromosome painting by ordering haplotypes in the spirit of the original Li and Stephens algorithm (Li and Stephens 2003). Namely, for each ordering *j*, we conduct the chromosome painting by conditioning donors of each recipient haplotype (*H*_2_, … ,*H_n_*) on the previous ones, such that
(1)
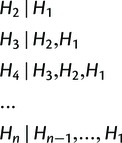

where (*H*_1_, *H*_2_, … , *H_n_*) is the ordered sample of *n* haplotypes. In typical applications, we conduct 10 random ordering and their reverse (i.e., *j* = 1, … , 20), which is justified in the Results section.

In each ordering, the chromosome painting gives posterior probability of donors for each polymorphic site on a recipient genome. For a given site *i*, this can be formatted as a matrix in which rows represent recipients and columns represent donors, with the values being normalized, so that each row sums up to 1. We call this the site-by-site copying probability matrix 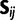 of site *i* and ordering *j*. By taking the average of the site-by-site copying probability matrix 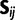 for all sites, an average copying probability matrix 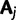 is calculated for ordering *j*. Two examples of the average copying probability matrices 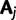 obtained for two different orderings (*j* = 1, 2) are shown in supplementary figure S1, Supplementary Material online. Because of the conditioning on donors of recombination, the matrices have values only in the lower triangles below the diagonals. Note that different orderings mean different conditioning of donors of recombination and give different site-by-site copying probability matrix 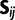 and average copying probability matrix 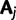.

Next, we examine the distance between a site-specific copying probability matrix 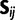 and the average copying probability matrix 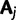. For each site *i* and each ordering *j*, we calculate *d_ij_* as the sum of squared distance of every element of 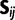 and 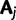:
(2)
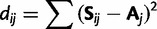

Then we calculate a distance statistic 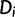 by taking the summation of *d_ij_* across orderings:
(3)
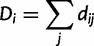



Intuitively, this distance statistic captures the extent of deviation of a specific site compared with the genome-wide average. We can also say that it captures the extent of genealogical changes due to recombination compared with the average genealogy. We expect atypical values (e.g., top percentile) of this statistic to indicate recombination hot regions or atypical sites, which are subject to frequent import. We also calculate bootstrap support of the distance statistic by resampling the contribution made to it by individual isolates in their role as recipients. Each strain still contributes to the statistic as a donor. Specifically, for each site *i* and each ordering *j*, we calculate 100 bootstrapped samples of *d_ij_* by resampling rows of 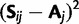 with replacement. This resampled version of the matrix is summed over all orderings. For each site *i,* we calculate the bootstrap support value as the number of bootstrapped samples that fall in the top percentile of the distribution of 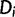 for all sites without bootstrapping.

### Visualization

To give an interpretable visualization of the ordered painting, we calculate the following matrix for each site of interest:
(4)
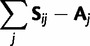



Namely, it is the matrix obtained by summation over all orderings of the differences between the site specific copying probability matrices 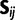 and the average copying probability matrices 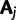. Positive values in the matrix indicate increased copying probabilities, whereas negative values indicate decreased copying probabilities compared with the genome-wide average.

### Application to Simulated and Real Complete Genome Sequence Data

We first applied the method to simulated data in the presence of recombination hot regions to test the sensitivity and specificity of inference of recombination hot regions. We used SimMLST (Didelot et al. 2009), which assumes a coalescent (Ancestral Recombination Graph [ARG]) with gene conversion model in a neutrally evolving unstructured closed population (Wiuf and Hein 2000). To generate data in the presence of recombination hot regions, we modified SimMLST, so that relative recombination rate can be specified for each simulated region. We simulated 100 genomes of 50 blocks of 5 kb each, in which there were two hot blocks with elevated recombination rate (hot vs. background ratio *α*). We used parameter values of 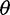 (mutation rate) = 2,500, 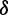 (mean tract length) = 542 bp, and 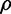 (background recombination rate) = 1,000. We assumed conditions of the hot versus background ratio *α* = 2, 3, 4, or 5 and conducted five runs of simulation for each condition. In each simulation run, we inferred recombination hot regions as blocks containing sites in the top percentile of the distance statistic values.

One factor which is known to influence methods to infer recombination is polymorphism levels (Posada and Crandall 2001; Posada et al. 2002). We also investigated variation in 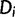 due to variation in mutation rates between genome regions. By using the modified version of simMLST, we simulated two sets of 100 genomes of 50 blocks of 5 kb each, in which there were two blocks with 5-fold higher mutation rate or half (25) of the blocks with 2-fold higher mutation rate. We also investigated the correlation between 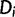 and polymorphism levels in the simulated data and another simulated data set, which had no variation in mutation or recombination rates. Nucleotide diversity at a polymorphic site was calculated by sliding windows implemented in VariScan version 2.0 (Hutter et al. 2006). We also applied the method to complete genomes of 27 *E. coli* isolates, which were recently analyzed by ClonalOrigin to reveal impact of recombination in shaping the genome evolution and diversification (Didelot, Méric, et al. 2012). We used the same nucleotide sequence alignment of 765 locally collinear blocks as used in the previous study.

### Application to Real Genome Sequence Data with Imputation

The method was also applied to genomes of 200 *C. jejuni* isolates in which recombination plays an important role (Wilson et al. 2009). One of them was a reference complete genome sequence (Parkhill et al. 2000) and the others were assembled de novo, resulting in an average of 77 contigs per genome (with a minimum of 15 and a maximum of 617). The 200 isolates were broadly sampled from 26 clonal complexes and various sources (supplementary table S1, Supplementary Material online). Allele sequences of the *C. jejuni* genomes were exported from BIGSdb (Jolley and Maiden 2010) by the default option that excludes truncated sequences. The exported data will be publicly available at Dryad, http://datadryad.org/ (last accessed March 7, 2014). Gene-by-gene alignments were conducted by MAFFT-EINSI (Katoh and Toh 2008), and we combined SNPs on each gene while preserving information of SNP positions to prepare genome-wide haplotype data.

Because of the larger amount of missing data in the sequences of *C. jejuni,* compared with *E. coli*, we conducted imputation for polymorphic sites with missing frequency 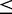
10% using BEAGLE (Browning BL and Browning SR 2009). In this case, after executing the chromosome painting algorithm, we calculated the average copying probability matrices 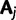 from the site-by-site copying probability matrices 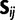 by masking columns and rows of missing individuals at site *i* and normalizing 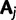
_,_ so that each row sums to 1. The masked copying probability matrix of each site was also normalized and used to calculate the distance statistic 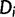.

## Results

### Accurate Inference of Recombination Hot Regions in Simulated Data

**Fig. 2.**
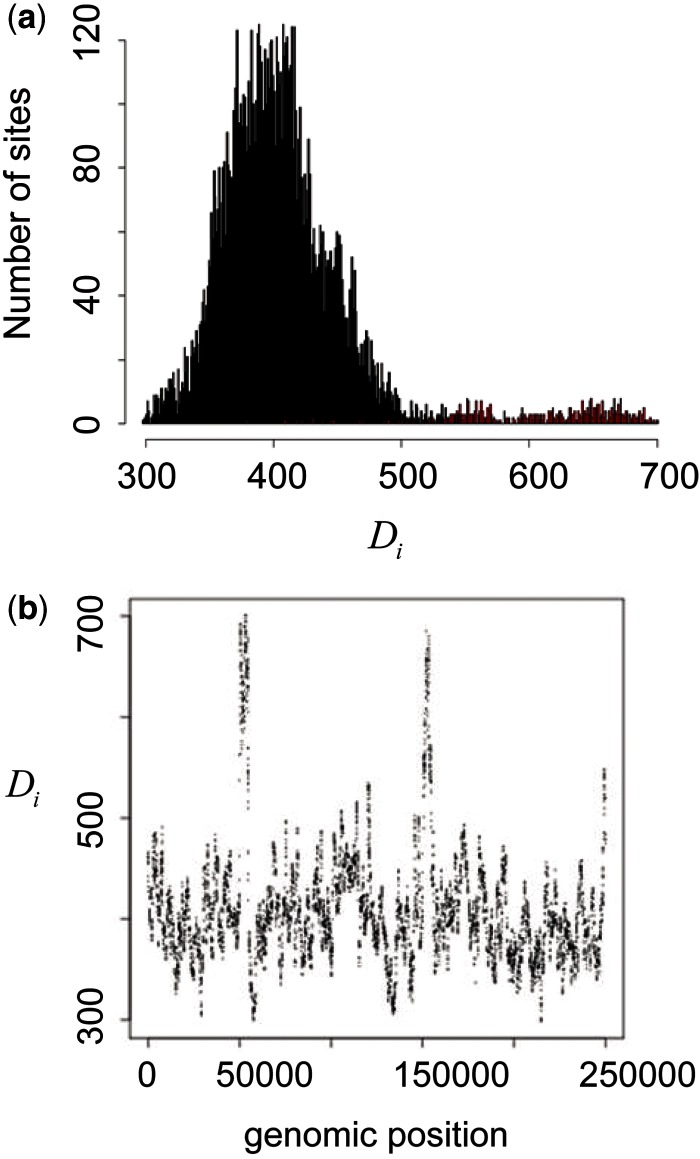
Inference of recombination hot regions in simulated data. An example with hot versus background ratio equal to 5 is shown. True recombination hot regions are located at 50,000–55,000 and 150,000–155,000. (*a*) Distribution of the distance statistic. (*b*) Plot of the distance statistic along the genome.

An example of distribution of the distance statistic 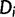 obtained from simulation with hot versus background ratio *α* = 5 is shown in figure 2. The statistic followed an approximately normal distribution for the background sites but was elevated for the two hot regions (which account for most of the values greater than 500 in fig. 2*a*, and peaks at 50,000 and 100,000 in fig. 2*b*). Sites within the top percentile of the distance statistic were all found in the two hot regions, and median bootstrap value among the atypical sites was 73 (interquartile range: 63–83).

**Fig. 3.**
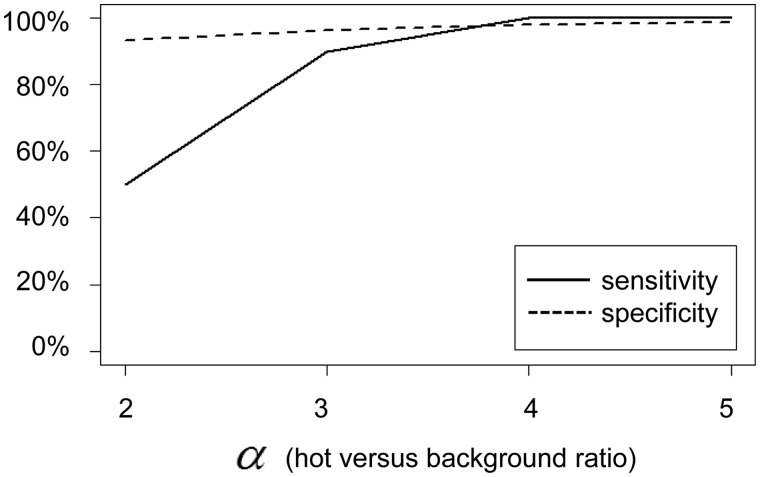
Benchmarking of inference of recombination hot regions. Sensitivity (solid) and specificity (dashed) are calculated from five different simulation runs, each of which assumes two true recombination hot regions (blocks) out of 50 blocks as shown in figure 2.

By conducting multiple runs of simulation and changing *α* from 2 to 5, we calculated sensitivity and specificity, assuming the sites with the top percentile of the distance statistic to be hot (fig. 3). The results indicate that the method is effective in distinguishing recombination hot regions and others when *α* is 4 or 5 (sensitivity of 100% and specificity of 98–99%). The method is less effective when *α* is 2 (sensitivity of 50% and specificity of 93%). An example of false inference under *α* = 5 is shown in supplementary figure S2, Supplementary Material online. The two highest peaks are located in true hot regions, but a third high peak was also detected. The median bootstrap value among sites in the false peak was 55 (interquartile range: 54–56).

We also examined two sets of simulated data, in which there were two blocks with 5-fold higher mutation rates or half of the blocks with 2-fold higher mutation rates. Distributions of 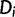 in the regions with elevated mutation rate and other regions are shown in supplementary figure S3*a* and *b*, Supplementary Material online. In either case, 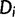 was not substantially elevated in the regions with elevated mutation rate.

We then examined a relationship between the distance statistic 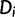 and nucleotide diversity per site in the simulated data, in the absence of variation in mutation rates. A result using sliding windows with 250 bp is shown in supplementary figure S4, Supplementary Material online. The plot shows weak correlation (0.15) between them and that the atypical sites with highest 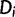 are not clustered in those with highest nucleotide diversity per site. We tried to use different windows sizes (from 500 bp to 1 bp), which account for all sites or only polymorphic sites, but results were consistent with the above one. When we examined another simulated data without variation in mutation or recombination rates, similar levels of correlation were found.

### Correlation with Results from ClonalOrigin

**Fig. 4.**
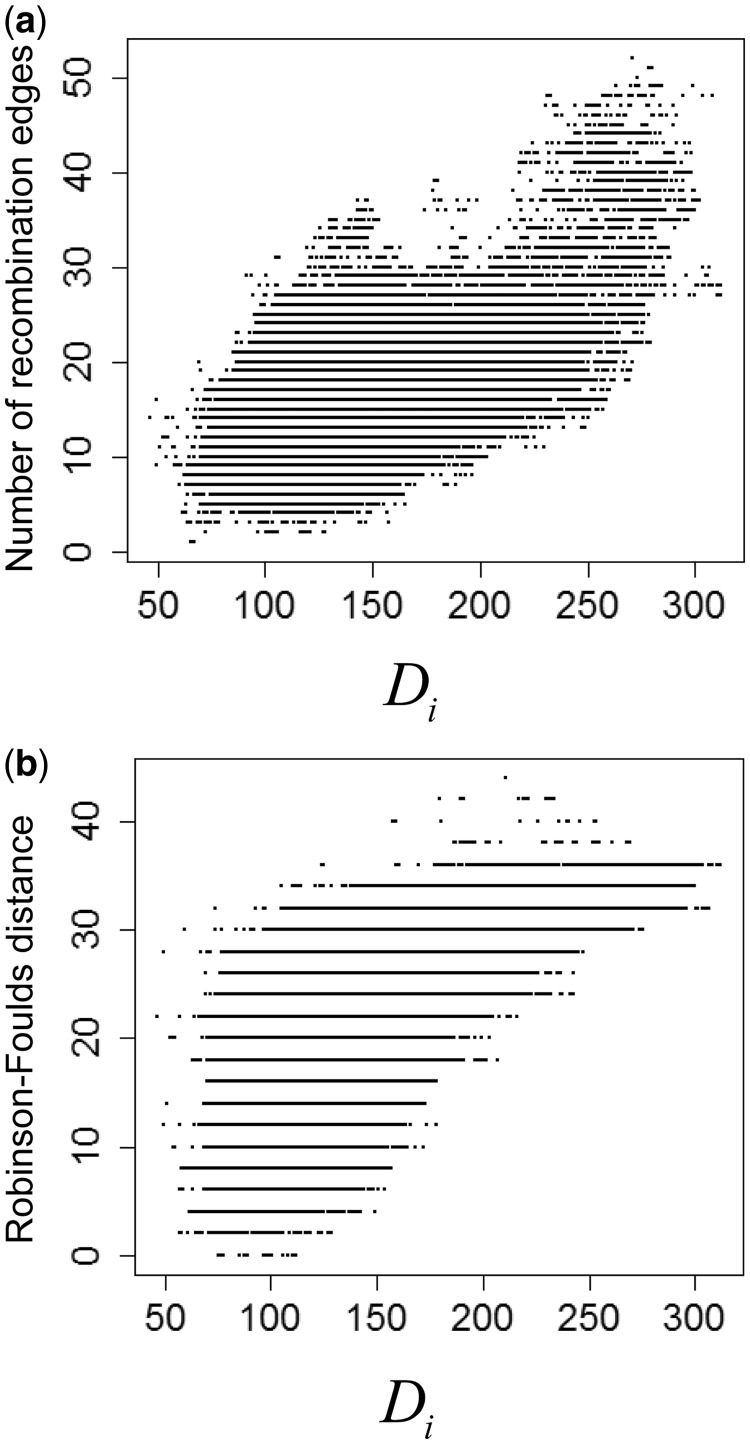
Correlation between the distance statistic and other measures of recombination in the ordered painting condition. Each dot indicates each polymorphic site in the *Escherichia coli* genomes. *X* axis is the distance statistic 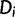 obtained from 10 different orderings and their reverse. (*a*) Correlation with the number of recombination edges of each site. (*b*) Correlation with Robinson–Foulds distances between the clonal genealogy and local tree of each site.

Next, we applied the ordered painting method to 27 *E. coli* genomes in which the clonal genealogy and recombination edges were previously inferred by ClonalFrame and ClonalOrigin (Didelot, Méric, et al. 2012). The results for 27 *E. coli* genomes are shown in figure 4.

When the number of recombination edges (events inferred by ClonalOrigin) of each site was used as a measure of the extent of recombination, the correlation coefficient was 0.53 (fig. 4*a*). The top right of figure 4*a* shows the sites with the highest values of the statistic and the number of recombination edges (>40 per site). The correlation coefficient was similarly high (0.59) when the Robinson–Foulds distance (Robinson and Foulds 1981) measuring differences in topology between a local tree of each site and the clonal genealogy is used as another measure of the extent of recombination (fig. 4*b*). The top right of figure 4*b* shows sites requiring more than 30 transformations to convert their local trees to the clonal genealogy. Those sites also show very high values for the distance statistic.

In figure 4, we applied the ordered painting method by doing 10 orderings and their reverse. Another result obtained by doing 100 orderings and their reverse is shown in supplementary figure S5, Supplementary Material online. The correlation coefficients were the same as when using 10 orderings and their reverse, which indicated that increasing the number of orderings did not improve the results. We also found that the values of the distance statistic obtained from two different sets of 10 orderings and their reverse were consistent (supplementary fig. S6, Supplementary Material online) with a correlation coefficient of 0.999. Therefore, a run of the ordered painting given 10 orderings and their reverse is sufficient to detect atypical sites. We caution, however, that the number of orderings required to get consistent results might differ between data sets, particularly if the composition is unbalanced, for example, due to many clones from a particular lineage being included in a sample. Therefore, the correlation between independent orderings should be tested by users exploring new data sets.

In the above results, we used orderings together with its reverse rather than independent orderings. This is because one ordering and its reverse had a correlation with the ClonalOrigin results significantly higher than two independent random orderings (*P* < 0.005, Wilcoxon rank sum test). Intuitively, opposite orderings are complementary because they are more likely to identify recombination events in different parts of the clonal genealogy than independent orderings are. The all-versus-all painting used in Lawson et al. gave lower correlations of 0.37 for the number of recombination edges of each site (supplementary fig. S7*A*, Supplementary Material online) and 0.42 for the Robinson–Foulds distance between local trees and the clonal genealogy (supplementary fig. S7*B*, Supplementary Material online). This indicates the ordered painting is more effective in capturing information on recombination.

**Fig. 5.**
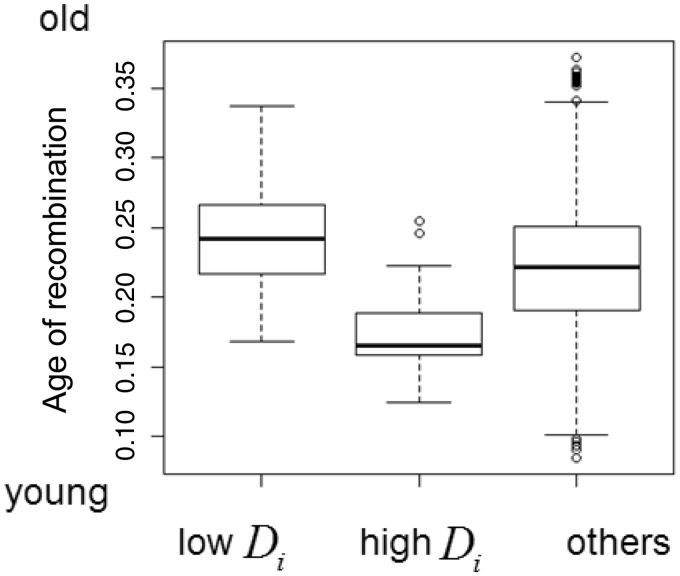
Comparison of average age of recombination edges on recipients among sites with intermediate number (20) of recombination edges. The sites are classified into the tree categories (from left to right): bottom 100 sites with low 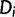, top 100 sites with high 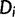, and others. The bold line indicates the median, and the bottom and top of the box indicate the 25th and 75th percentiles, respectively.

The above results show that there is a high correlation between the distance statistic and the measures of the extent of recombination inferred by ClonalOrigin. However, for sites with an intermediate number of recombination edges or level of Robinson–Foulds distance, the values of the distance statistic are widely distributed from low to high. We therefore focused on the 100 sites with lowest or highest values of the distance statistic among sites with an intermediate number (20) of recombination edges and examined the differences between them. We found that the average recombination recipients estimated by ClonalOrigin were significantly younger in the sites with highest values of the distance statistic than in those with the lowest values (fig. 5, *P* < 10^−^^15^, Wilcoxon rank sum test). This indicates that as expected, the distance statistic is better at detecting relatively recent recombination events.

### Visualization of Genome-Wide Average Matrix

Although it does not capture all of the available information on recombination events, the fineSTRUCTURE algorithm clusters the isolates appropriately based on the whole genome painting and this clustering is useful in visualizing and interpreting results. A result of fineSTRUCTURE on the *E. coli* isolates is shown in supplementary figure S8, Supplementary Material online. The 27 *E. coli* isolates are assigned into 11 subgroups. The subgroups are seen along diagonal blocks in the coancestry matrix which summarizes the expected number of chunks of DNA imported from a donor to a recipient genome.

**Fig. 6.**
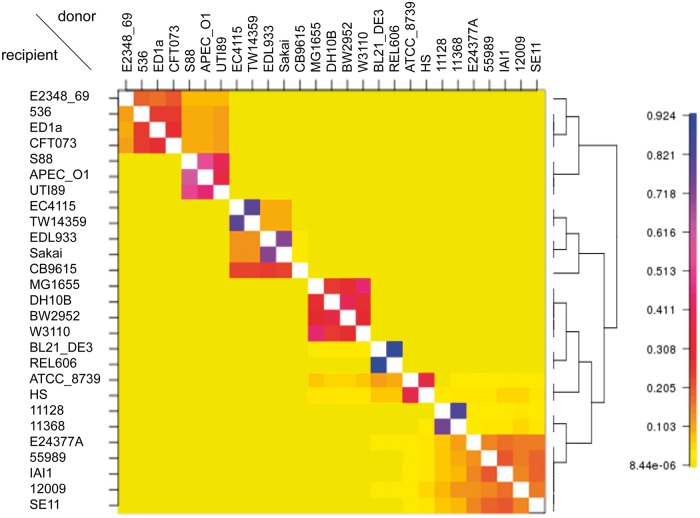
Average copying probability matrix in the all-versus-all painting condition. The right is a tree inferred by fineSTRUCTURE.

In the case of the all-versus-all painting, the average copying probability matrix can be also calculated (fig. 6). It seems to have similar information content to the coancestry matrix of fineSTRUCTURE (supplementary fig. S8, Supplementary Material online). If the bacteria are independently sampled from a freely recombining population, then the coancestry matrix of fineSTRUCTURE or the average copying probability matrix in the all-versus-all painting will be approximately flat. Because our method is dependent on finding deviations from genome-wide patterns of descent, a data set of equally related strains will provide little or no information on hot regions. Therefore, either of the two genome-wide average matrices, namely the coancestry matrix of fineSTRUCTURE or the average copying probability matrix in the all-versus-all painting, should be checked to see that there are actually clonally related strains in the data set before proceeding with further analysis.

### Visualization of Individual Sites

**Fig. 7.**
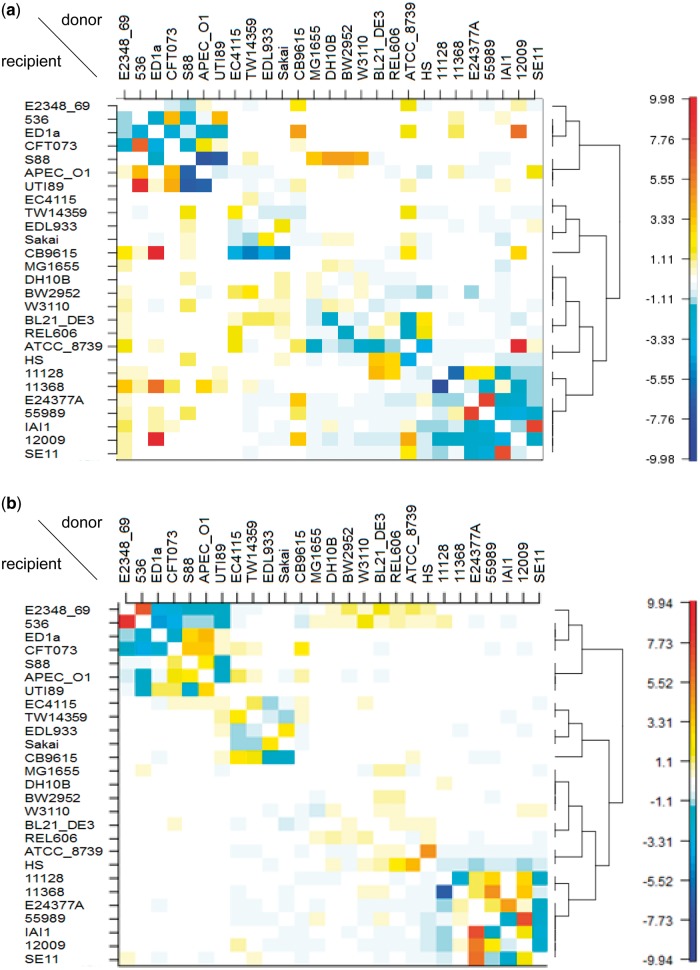
Visualization of deviation of the extent of recombination from the genome-wide average. The value of each cell of the matrix is obtained from equation (4): summation of a site-specific copying probability matrix minus average copying matrix across ten different orderings and their reverse. The name of each strain is indicated on the left and top. The right is a tree inferred by fineSTRUCTURE. (*a*) An atypical site with the highest level of recombination. (*b*) A typical site with the intermediate level of recombination.

In the results above, the atypical sites were detected as those with the highest values (top percentile) of the distance statistic. These sites have the largest distances from the average copying matrices (supplementary fig. S1, Supplementary Material online) across the 10 orderings and their reverse. The deviation is visualized using equation (4) as a matrix, and an example is shown in figure 7. The matrix of an atypical site (position 2092621 on *hisH* in the reference genome K-12 MG1655) is different from that of a typical site with an intermediate value of the distance statistic. The number of red and orange cells with increased copying probabilities is higher for the atypical site than for the typical site. The red and orange cells are frequently found far from the diagonal, suggesting some recombination events occurred between the subgroups identified by fineSTRUCTURE. The number of recombination edges of this atypical site is 47, which is high and within top 0.1% (fig. 4). We have confirmed that a different set of 10 random orderings and their reverse showed visually similar result for these sites although individual cells do have different values (supplementary fig. S9, Supplementary Material online).

### Atypical Sites and Recombination Hot Regions

**Fig. 8.**
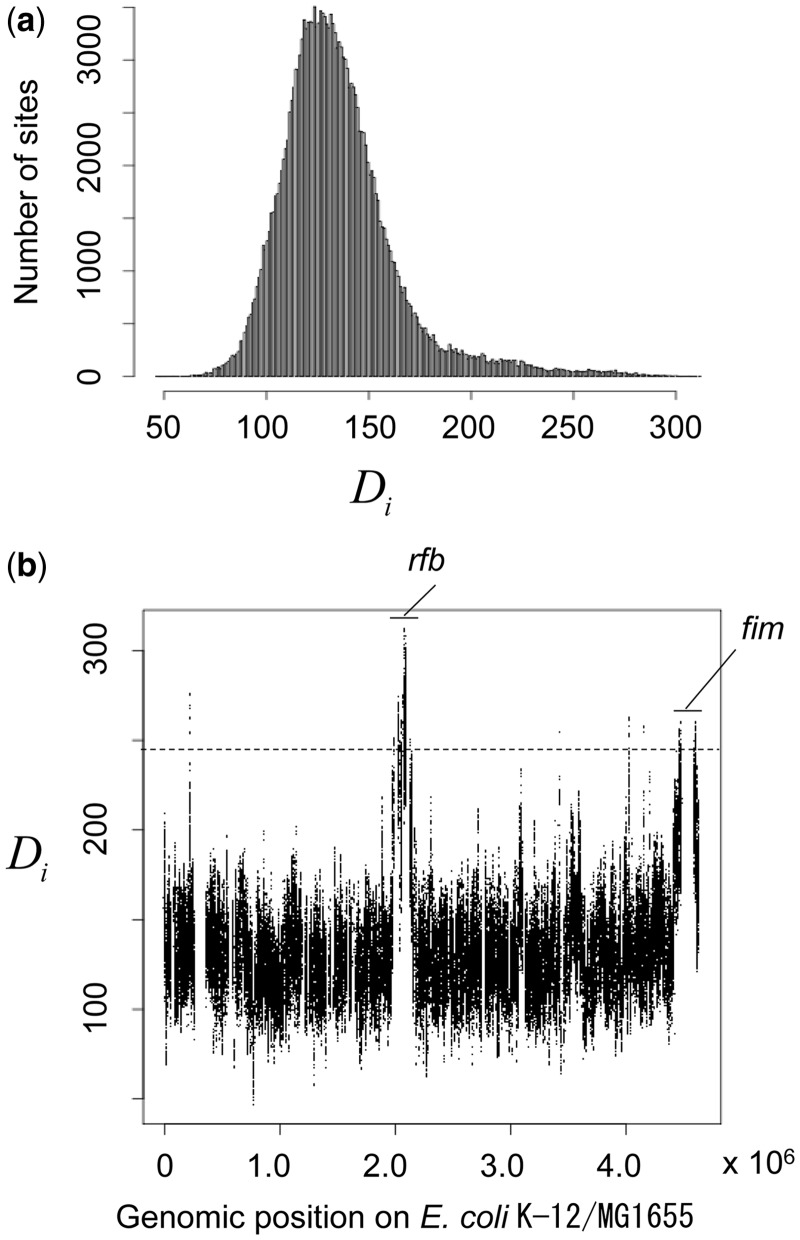
Results for the 27 *Escherichia coli* genomes. A total of 190,551 SNPs were used. (*a*) Empirical distribution of the distance statistic. (*b*) The relative intensity of recombination along the genome. The *X* axis indicates the position in the reference genome K-12 MG1655 (Blattner et al. 1997). *Y* axis indicates the value of the distance statistic. The dotted line represents the top percentile. Two large regions are indicated by names of loci as a symbol.

The empirical distribution of the distance statistic for *E. coli* (fig. 8*a*) has a long tailed distribution similar to that obtained by simulation in the presence of recombination hot regions (fig. 2*a*). Most of the atypical sites above a threshold (top percentile) were clustered in two large regions corresponding to *rfb* and *fim* (fig. 8*b*), which have previously been reported as hotspots for recombination or phylogenetic incongruence (Touchon et al. 2009; Didelot, Méric, et al. 2012). The median bootstrap value among these atypical sites was 86 (interquartile range: 71–95). All of the other atypical sites were located in or close to ribosomal genes: three genes of 23S ribosomal RNA—B0204, B3275, and B3854 in the K-12 MG1655 genome, and two genes of 16S ribosomal RNA—B3851 and B3968.

**Fig. 9.**
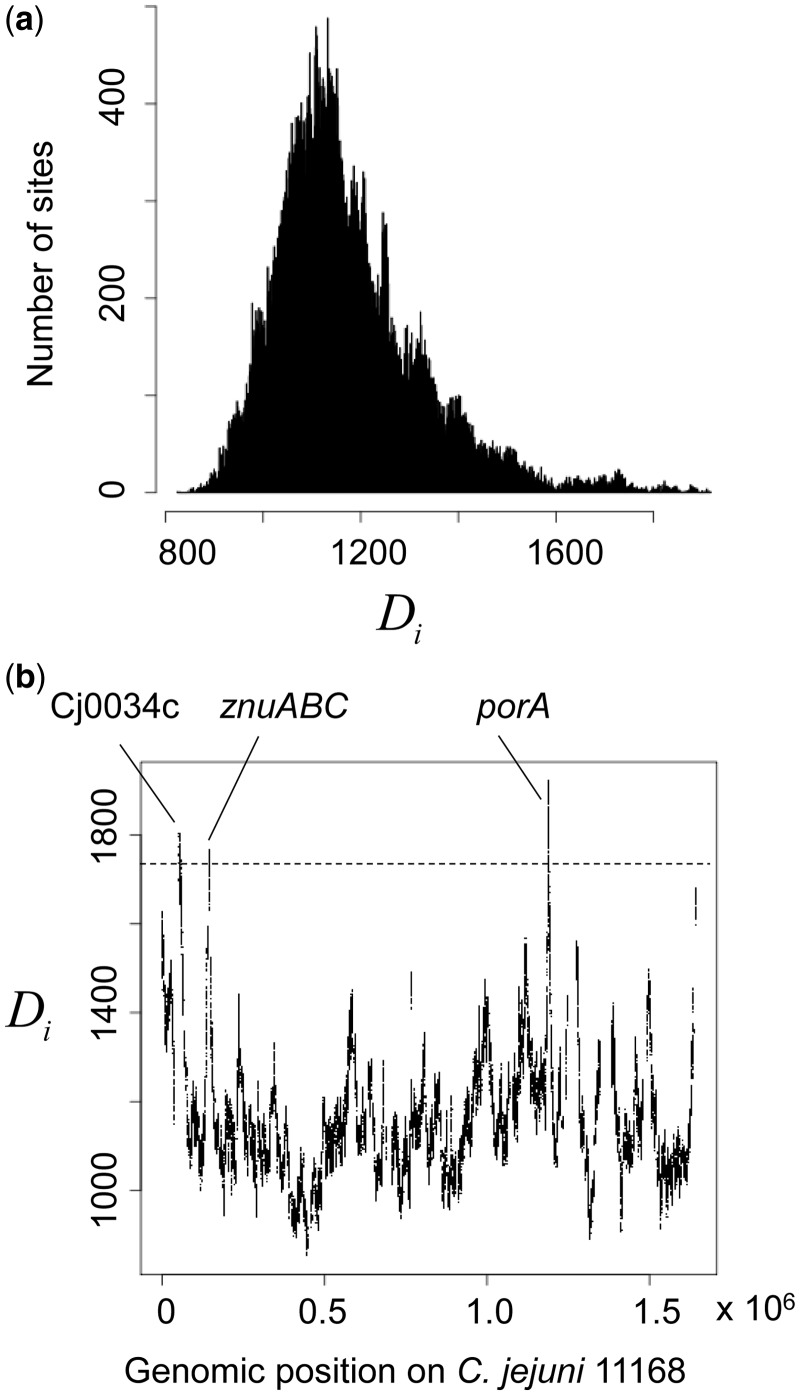
Results for the 200 *Campylobacter jejuni* genomes. The format is the same as figure 8. A total of 112,253 SNPs were used. (*a*) Empirical distribution of the distance statistic. (*b*) The relative intensity of recombination along the genome. The *X* axis indicates the position in the reference genome NCTC11168 (Parkhill et al. 2000).

The method was also applied to 200 genomes of *C. jejuni* in which recombination hot regions have not been previously described. The coancestry matrix of fineSTRUCTURE is shown in supplementary figure S10, Supplementary Material online, which shows much of the matrix is concentrated near to the diagonal, and therefore contains information of clonal relationships among the isolates. The method indeed revealed recombination hot regions (fig. 9). Furthermore, the distribution of the distance statistic shows a long tail (fig. 9*a*), also suggesting the presence of recombination hot regions. The atypical sites in the top percentile above the threshold were clustered in three regions (fig. 9*b*). Median bootstrap value among these atypical sites was 87, with an interquartile range of 73–100.

A list of genes contained in the hot regions is given in table 1. They are classified into the three regions in the reference genome: 51967–57211 (Cj0034c–Cj0038); 143953–146506 (Cj0141c–Cj0143c); and 1188414–1191631 (Cj1258c–Cj1260c). By using the names of representative loci, these three regions are indicated in figure 9*b* as Cj0034, *znuABC*, and *porA*, respectively. Interestingly, about half of the genes are related to membrane proteins, which is significantly higher (*P* < 0.0005, Fisher’s exact test) than in the whole genome.

**Table 1.** Genes in Inferred Recombination Hot Regions in *Campylobacter jejuni*.

Locus Tag^a^	Position^a^	Description
Cj0034c	51967–52668	Putative periplasmic protein, probable integral membrane protein, containing six possible transmembrane domains in C-terminal half, supporting host cell adhesion
Cj0035c	52665–53867	Putative efflux transporter belonging to the major facilitator super family
Cj0036	53970–55319	Hypothetical protein
Cj0037c	55343–56386	Putative cytochrome c
Cj0038c	56564–57211	Hypothetical protein (possible membrane protein, containing two possible transmembrane domains at the N-terminus)
Cj0141c	143953–144756	*znuB*, integral membrane protein in ZnuABC system^b^
Cj0142c	144749–145603	*znuC*, ATPase component in ZnuABC system^b^
Cj0143c	145616–146506	*znuA*, zinc-binding protein in ZnuABC system^b^
Cj1258	1188414–1188869	Possible phosphotyrosine protein phosphatase
Cj1259	1189121–1190395	*porA*, major outer membrane protein (MOMP)
Cj1260c	1190510–1191631	*dnaJ*, chaperone

^a^The *C. jejuni* 11168 genome.

^b^ZnuABC zinc update system, part of the family of ABC transporters.

The first region (Cj0034c–Cj0038c) overlaps with previously reported hypervariable regions (C0032–Cj0036) (Parker et al. 2006) in which three genes related to membrane proteins (Cj0034c, Cj0035c, and Cj0038c) are located. Cj0034c was recently reported to be an adhesion and virulence factor in *C. jejuni* (Nielsen et al. 2011). Cj0035c encodes a putative efflux pump (membrane transporter) belonging to the major facilitator family. Cj0038c is a hypothetical gene that encodes a possible membrane protein containing two possible transmembrane domains at the N-terminus. The second region (Cj0140–Cj0143c) encodes a zinc-dependent operon of ZnuABC zinc uptake system, part of the family of ABC transporters. The ZnuABC system is used by a number of bacterial pathogens as an essential factor for host colonization and virulence (Davis et al. 2009). The third region (Cj1258c–Cj1260c) includes the *porA* gene, which encodes the major outer membrane protein (MOMP). The MOMP is a porin, which is responsible for adhesion to the intestinal mucosa (Moser et al. 1997).

Inaccuracies in imputation might inflate values of the distance statistic compared with cells without missing data. To minimize the effect of imputation on the distance statistic, we prepared the site-by-site copying probability matrix 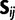 by masking columns and rows for which the individuals had missing data and then calculated the average copying probability matrix 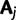. For each site *i*, we then calculated the distance statistic.

To examine whether the inferred recombination hot regions in *C. jejuni* are not artifacts of the imputation method used, we created bins of SNPs sorted by missing frequency (10 SNPs/bin). Average values of the distance statistic of the SNP bins are shown in supplementary figure S11, Supplementary Material online, based on using polymorphic sites with missing frequency 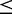
10%, which indicates no overall relationship between missing frequency and the distance statistic. The atypical sites with high values of the distance statistic are not collocated with those with high missing frequency. However, if sites with up to 50% missing data are included, many of these additional sites have atypically high values for the distance statistic (supplementary fig. S12, Supplementary Material online).

When sites with missing frequency 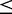
10% are imputed (supplementary fig. S13*A* and *B*, Supplementary Material online), the contribution of masked cells is indeed inflated (supplementary fig. S13*B*, Supplementary Material online) compared with the other cells (supplementary fig. S13*A*, Supplementary Material online). When sites with missing frequency 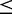
50% are imputed (supplementary fig. S13*C* and *D*, Supplementary Material online), the inflation is seen not only in the masked cells (supplementary fig. S13*D*, Supplementary Material online) but also in other cells (supplementary fig. S13*C*, Supplementary Material online). This explains why the inflation was seen in supplementary figure S12, Supplementary Material online, even after the masking was conducted.

## Discussion

We developed a computationally efficient method for identifying recombination hot regions. The method is based on the chromosome painting algorithm, which is implemented in ChromoPainter (Lawson et al. 2012). The input data are aligned bacterial genome sequences, as for ChromoPainter. If there are sites with missing data, then they need to be either imputed or excluded. We have found that in practice it is necessary to exclude sites with >10% missing data. Here, we describe and implement programs to apply ChromoPainter with different random orderings and to conduct postprocessing of the data for calculating and visualizing statistics of interest. Many of the steps in the pipeline are parallelizable and computation of the ordered painting of the 27 *E. coli* genomes is complete within approximately half an hour using about 100 CPU cores, whereas ClonalOrigin took almost a month on hundreds of CPU cores. The analysis of 200 *C. jejuni* genomes was more time consuming but was also complete within several hours. A package of programs called orderedPainting is publicly available at https://github.com/bioprojects/orderedPainting (last accessed March 7, 2014).

We applied the ordered painting method first to simulated data in which the majority of the genome had a background rate, whereas others were in hot regions, with a factor *α* more recombination. The method reliably identified the hot regions when *α* was 4 or higher and had some power when *α* was 2 or 3. We then applied the ordered painting method to the *E. coli* genome data, which were previously analyzed by ClonalOrigin, and confirmed previously known hotspots of phylogenetic incongruence or recombination (*rfb* and *fim*, fig. 8). Although both of the methods showed the consistent results in terms of inference of recombination hot regions, we would like to discuss and clarify theoretical differences between them below.

ClonalOrigin can be considered as a “gold standard” for inferring recombination in bacteria because it identifies a donor and recipient for each recombination event in the ancestry of the sample and the time at which the event occurred. Conceptually, the donor and recipient can be thought of as individual bacteria, living at a particular time in the past. Within the algorithm, the relationships of these bacteria to those that have been sampled are represented by positions in the clonal genealogy; the higher up in the tree, the further back in time the recombination event occurred. This can be considered as the extent of what can be inferred from the full ARG (Wiuf and Hein 2000; Hein et al. 2005) and is essentially all the information that sequence data can provide on historical recombination events. A hot region is a part of the genome where there is a high rate of recombination events that impact on the clonal genealogy. This information can be summarized from the ClonalOrigin output by the number of recombination edges that affect each site.

ChromoPainter detects shared ancestry and changes in it along the genome and thus can also be considered to be mining information about the ARG based on genome sequences. The algorithm works by reconstructing the genome of each bacterium in the sample as a mosaic of all the other bacteria in the sample. To identify recombination hot regions, we have attempted to extract the most relevant information on departures from clonal descent provided the ChromoPainter output. Specifically, a recombination event at a particular site is likely in the ancestry of a bacterium if the donor for the site inferred by ChromoPainter is infrequently used as a donor for that individual elsewhere in the genome-wide painting. The genome-wide painting will much of the time reflect patterns of clonal descent. Our statistic for deviations from the genome wide average is summed across both donors and recipients. In practice, we have found it is strongly correlated with the number of recombination edges found by ClonalOrigin and particularly reflects recombination events in the relatively recent history of the sample.

It is possible in principle to try and infer additional information on recombination from the ChromoPainter results, although we do not attempt this here. For example, a high rate of ancestry switches is likely to correlate with the frequency of recombination start and end point and so might be used to identify hotspots, similar to the original application of Li and Stephens (2003). Further, the painting does provide some information on which donor and recipient are likely to be involved in particular recombination events. However, this information is likely to be more difficult to interpret than, for example, the output of ClonalOrigin because of the order dependence of the algorithm and the absence of a tree within the algorithm to provide a clonal context.

As well as being computationally intensive, ClonalOrigin requires a fully resolved clonal genealogy to be estimated. This is a difficult computational problem in itself and becomes problematic or impossible when there are isolates in the data set that have little or no shared clonal frame. The painting method can be applied to arbitrary data sets and should be able to extract a signal as long as “some” isolates have clonal relatives. However, if isolates are unrelated members of a freely recombining population, then the genome wide average copying matrix will be flat, that is to say, that every cell in the matrix has similar value. Under these circumstances, the ordered painting method will not extract a signal. For most bacterial species, there is enough clonal structure within global samples that this will not be an issue.

There are many other statistical methods for studying recombination based on sequences, but few of them are suited to the specific problem of identifying hot regions. Many of the methods used in eukaryotes are based on the four-gamete test of recombination (Hudson and Kaplan 1985; McVean et al. 2002; McVean et al. 2004; Chan et al. 2012) and examine pairs of SNPs, to identify the rate at which genealogical ancestry changes due to recombination. These methods are suited to identifying hotspots rather than hot regions. Li and Stephens (2003) studied hotspots based on using the copying algorithm to identify high rates of ancestry switching. Meanwhile, BratNextGen (Marttinen et al. 2012) identifies recombination events within a clonal context but without inferring the number of recombination events at each locus.

Our comparison with ClonalOrigin for the *E. coli* analysis implies that our method is less sensitive to old recombination events (many of which will be shared by many strains) than to new ones. Figure 1 provides an explanation for this. Even if a recombination occurred in the common ancestor of two or more strain in the sample, it is unlikely to be detected more than once and may not be detected at all for particular orderings.

A known confounder of recombination rate estimators such as homoplasy is variation in polymorphism levels (Posada and Crandall 2001; Posada et al. 2002; Yahara et al. 2012). We found that in simulated data, variation in mutation rates between regions had little effect on values of 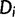 (supplementary fig. S3, Supplementary Material online). We found a positive correlation between 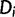 and polymorphism levels in simulated data with or without variation in recombination or mutation rates. Correlations can arise due to variation in genealogical history, because recombination changes the genealogy and thus changes the amount of polymorphism that is observed. The direction and amount of this effect are likely to depend in a complicated way on both the composition of the sample and patterns of gene flow. We conclude that our method is not substantially confounded by variation in polymorphism levels and that application of statistical methods 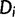 to correct for confounding would be complicated by the risk of removing real correlations between recombination and polymorphism that may be present in the data.

Our method, like others based on patterns of within population variation, detects recombination events that have survived in the population. Therefore, natural selection will inevitably affect inferences. One factor that can systematically alter the amount of recombination and diversity that is observed is diversifying selection. Correlations between 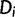 and nucleotide diversity in the real data of *E. coli* and *C. jejuni* are shown in supplementary figure S14, Supplementary Material online. In the case of *C. jejuni*, the region of the genome with the highest 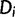 values was the *porA* locus, which also had exceptional levels of polymorphism (supplementary fig. S14*b*, Supplementary Material online) and is a likely target of diversifying selection, although the other membrane proteins detected by our method were not exceptionally diverse. In our view, it is very challenging to disentangle the effect of natural selection from other causes of variation in observed recombination rates based on the pattern of variation at individual loci.

Another potential confounder is a recombination imported from a very distant source. We also examine its effect on 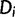 by artificially incorporating a distant sequence to the simulated data with elevated recombination rate. The result is shown in supplementary figure S15, Supplementary Material online. 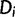 is not elevated, and the true recombination hot regions are inferred as previously.

In the application of the ordered painting method to the *E. coli* data set, we also found that a minority of the atypical sites were located in ribosomal genes in five different locations of the *E. coli* genome. However, when the number of genome sequences is increased, these signals disappear and these genes were also not found in 10 independent 27 *E. coli* data sets. The presence of hot regions at these genes is therefore not well supported, although the hint that there may be higher recombination rates at ribosomal genes is intriguing.

Applying the ordered painting method to 200 *C. jejuni* genomes revealed three recombination hot regions (Cj0034c, *znuABC* and *porA*). Genes related to membrane proteins are significantly more frequent in these regions. Frequent recombination in these genes presumably promotes diversification of the proteins that are advantageous for host interaction and colonization and are likely to be under diversifying selection due to the action of the host immune system. Recent experiments showed that inactivation of Cj0034c dramatically reduced the ability of *C. jejuni* to adhere to the intestinal epithelial cell line (Nielsen et al. 2011). Another experimental study reported that *znuABC* in *C. jejuni* is essential for colonization of the chicken ceaca and for growth in low-zinc environments (Davis et al. 2009).

Among the other genes related to membrane proteins, *porA* is notable for being extremely genetically diverse (Cody et al. 2009) and has been used as a tool for antigen gene sequence typing (Colles and Maiden 2012). So far, about 1,400 *porA* nucleotide and MOMP (PorA) peptide sequences are known and registered in the public database (http://pubmlst.org/campylobacter/, last accessed March 7, 2014) (Colles and Maiden 2012; Sheppard, Jolley et al. 2012). The high diversity among *porA* alleles is indicative of strong positive immune selection (Colles and Maiden 2012) and a previous study presented evidence of an excess of nonsynonymous substitutions in the putative surface-exposed loops (Cody et al. 2009).

Cj0035c encodes a putative efflux pump (membrane transporter), which is regulated by a transcriptional repressor CmeR modulating the expression of the multidrug efflux pump CmeABC (Guo et al. 2008). It is annotated as similar to the BCR_ECOLI bicyclomycin resistance protein. The gene may be related to drug resistance. Besides the genes related to membrane proteins, Cj0037c is a gene that encodes an uncharacterized cytochrome C related to stress survival of *C. jejuni* (Gaynor et al. 2005). This has been reported to show significantly higher expression in a laboratory strain adapted for survival in higher O_2_ conditions. Its frequent recombination could contribute to the survival of *C. jejuni* in stressful environments.

The ordered painting method that we have introduced here will be applicable to identify recombination hot regions in other important bacterial species. That will improve our understanding of recombination landscape across the bacterial genome, distribution of recombination hot genes in nature, and the association of recombination with the emergence of important phenotypes such as pathogenicity.

## Supplementary Material

Supplementary figures S1–S15 are available at *Molecular Biology and Evolution* online (http://www.mbe.oxfordjournals.org/).

Supplementary Data
