# Supplementary material for: Efficient Inference of Recombination Hot Regions in Bacterial Genomes
Source: Mol Biol Evol. 2014 Feb 27;31(6):1593–605. doi: 10.1093/molbev/msu082 (PMC4032127; doi:10.1093/molbev/msu082)
Supplement: Supplementary Data [file supp_31_6_1593__index.html]

Efficient inference of recombination hot regions in bacterial genomes — Efficient Inference of Recombination Hot Regions in Bacterial Genomes — Efficient Inference of Recombination Hot Regions in Bacterial Genomes — Supplementary Data 

# Efficient Inference of Recombination Hot Regions in Bacterial Genomes

## Supplementary Data

files

**Files in this Data Supplement:**

- Supplementary Data - pdf file
