# Supplementary material for: Efficient Inference of Recombination Hot Regions in Bacterial Genomes
Source: Mol Biol Evol. 2014 Feb 27;31(6):1593–605. doi: 10.1093/molbev/msu082 (PMC4032127; doi:10.1093/molbev/msu082)
Supplement: Supplementary Data [file supp_msu082_Full_Manuscript_SI_Figs_combined_with_Legends_TableS.pdf]

Figure Legends

Figure S1. Examples of average copying probability matrices. (a) An example obtained from an ordering. (b) Another example obtained from another ordering.

Figure S2. An example of false inference. The dotted line represents the top percentile. The hot versus background ratio is equal to 5. True recombination hot region regions are located at 50000-55000 and 150000-155000**.**

Figure S3. Variation in the distance statistic due to variation in mutation rates between genomic regions. (a) Simulated data with 2 out of 50 blocks with 5 fold higher mutation rate. (b) Simulated data with 25 out of 50 blocks with 2 fold higher mutation rate.

Figure S4. Correlation between the distance statistic and nucleotide diversity per site in the simulated data.

Figure S5. Correlation between the distance statistic and other measures of recombination in the ordered painting condition. The format of the figure is the

same as Figure 4. X-axis is the distance statistic D_i obtained from 100 different orderings and their reverse. (a) Correlation with the number of recombination edges of each site. (b) Correlation with distances between the clonal genealogy and local tree of each site.

Figure S6. Correlation between the distance statistic obtained from two set of 10 different orderings and the opposite.

Figure S7. Correlation between the distance statistic and other measures of recombination in the “all-versus-all” painting condition. The format is the same as Figure 4. (a) Correlation with the number of recombination edges of each site. (b) Correlation with distances between the clonal genealogy and local tree of each site.

Figure S8. A result of fineSTRUCTURE of the *E. coli* isolates. The colour of each cell of the matrix indicates the expected number of chunks imported from a donor genome (column) to a recipient genome (row). The name of each strain is indicated on the left and top. The tree in the right shows clustering for assignment of population subgroups.

Figure S9. Visualization of deviation of the extent of recombination from the genome-wide average. The format of the figure is the same as Figure 7. This is a result obtained from another 10 different orderings and the opposite for the same sites as in Figure 7. (a) An atypical site with the highest level of recombination. (b) A typical site with the intermediate level of recombination.

Figure S10. Co-ancestry matrix of the *C.jeuni* isolates.

Figure S11. Relation between missing frequency and the distance statistic. Each vertical bar corresponds to a bin of SNPs (10 SNPs per bin) sorted by missing frequency. The Y-axis represents the average value of the distance statistic per bin.

Figure S12. Relation between missing frequency and the distance statistic when sites with missing frequency $\leq$ 50% are imputed. Each vertical bar corresponds to a bin of SNPs (10 SNPs per bin) sorted by missing frequency. Y-axis: average value of the distance statistic per bin.

Figure S13. Inflation of the distance measure by imputation. A-B are results when sites with missing frequency $\leq$ 10% are imputed, while C-D are those when sites with missing frequency $\leq$ 50% are imputed. X-axis: values of $(\mathbf{S}_{ij} - \mathbf{A}_j)^2$ averaged over cells with (A, C) or without (B, D) missing data for each site. Y-axis: number of sites.

Figure S14. Correlation between the distance statistic and nucleotide diversity per site in the real data. Sliding windows with 250bp were used to calculate nucleotide diversity per site. (a) The *E. coli* data. Correlation coefficient is 0.09. (b) The *C. jejuni* data. Correlation coefficient is 0.21.

Figure S15. Inference of true recombination hot regions in the presence of a distant recombination. A distant sequence is artificially imported to a region (5001-10000) in the simulated data, in which D_i is not elevated.

Figure S1

a.

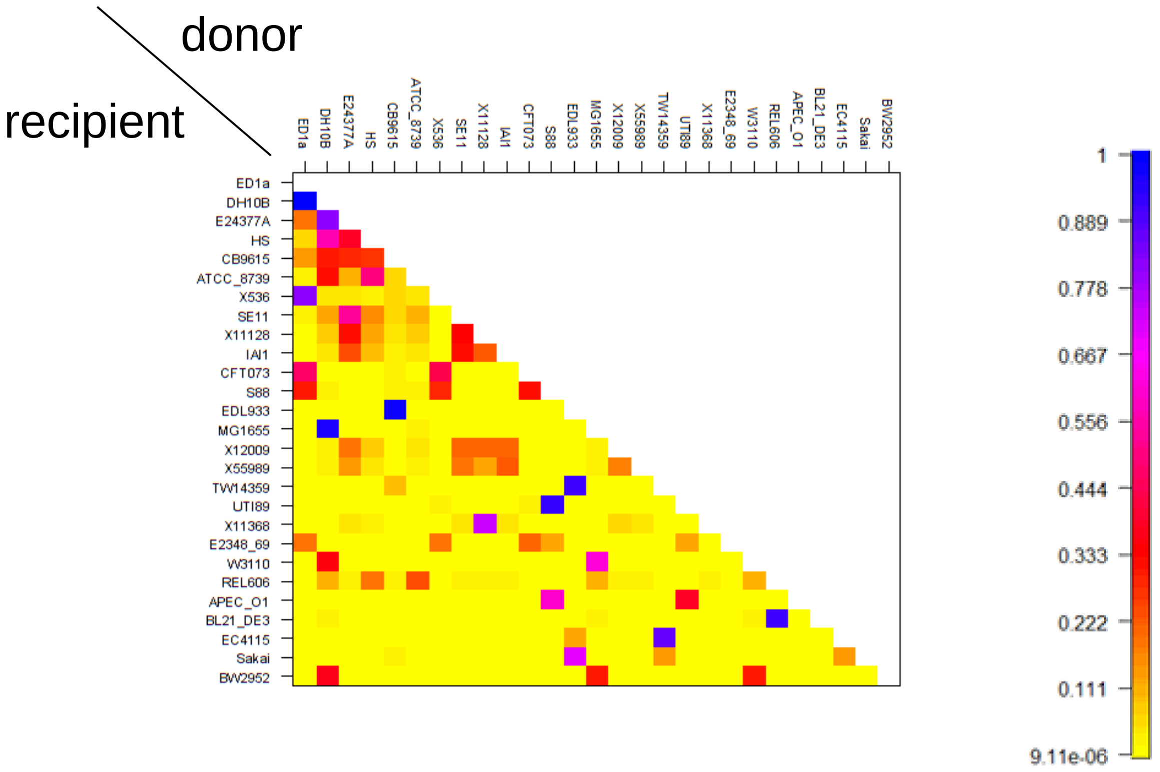
donor
recipient
ED1a
DH10B
E24377A
HS
CB9615
ATCC_8739
X536
SE11
X11128
IAI1
CFT073
S88
EDL933
MG1655
X12009
X55989
TW14359
UTI89
X11368
E2348_69
W3110
REL606
APEC_O1
BL21_DE3
EC4115
Sakai
BW2952
1
0.889
0.778
0.667
0.556
0.444
0.333
0.222
0.111
9.11e-06

b.

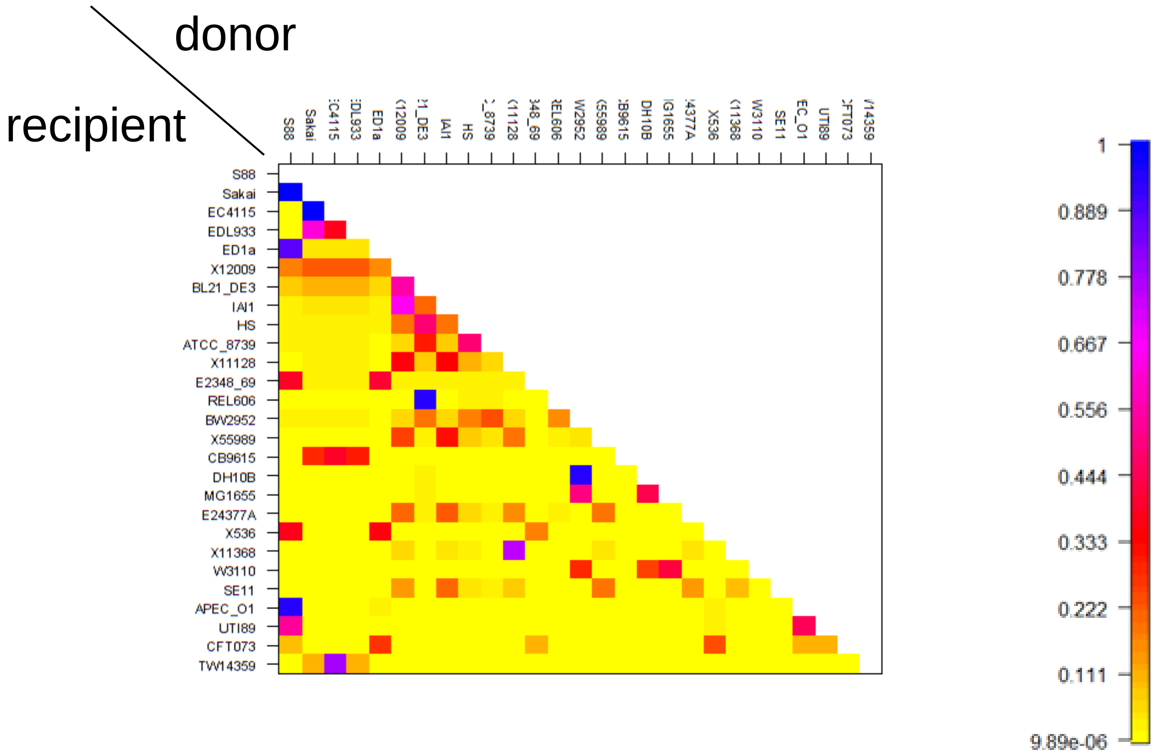
donor
recipient
S88
Sakai
EC4115
EDL933
ED1a
X12009
BL21_DE3
IAI1
HS
ATCC_8739
X11128
E2348_69
REL606
BW2952
X55989
CB9615
DH10B
MG1655
E24377A
X536
X11368
W3110
SE11
APEC_O1
UTI89
CFT073
TW14359
1
0.889
0.778
0.667
0.556
0.444
0.333
0.222
0.111
9.89e-06

Figure S2

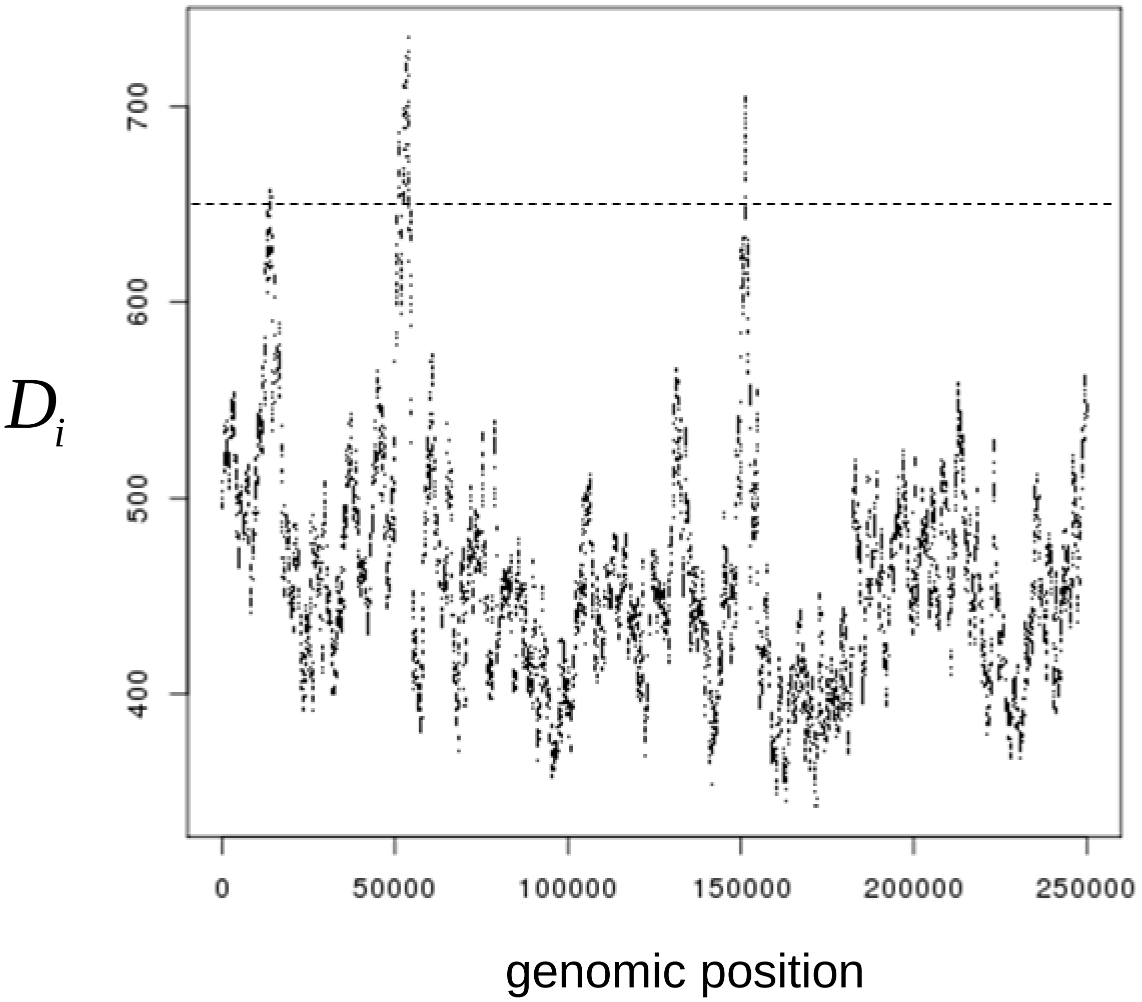
700
600
500
400
D_i
0
50000
100000
150000
200000
250000
genomic position

a.

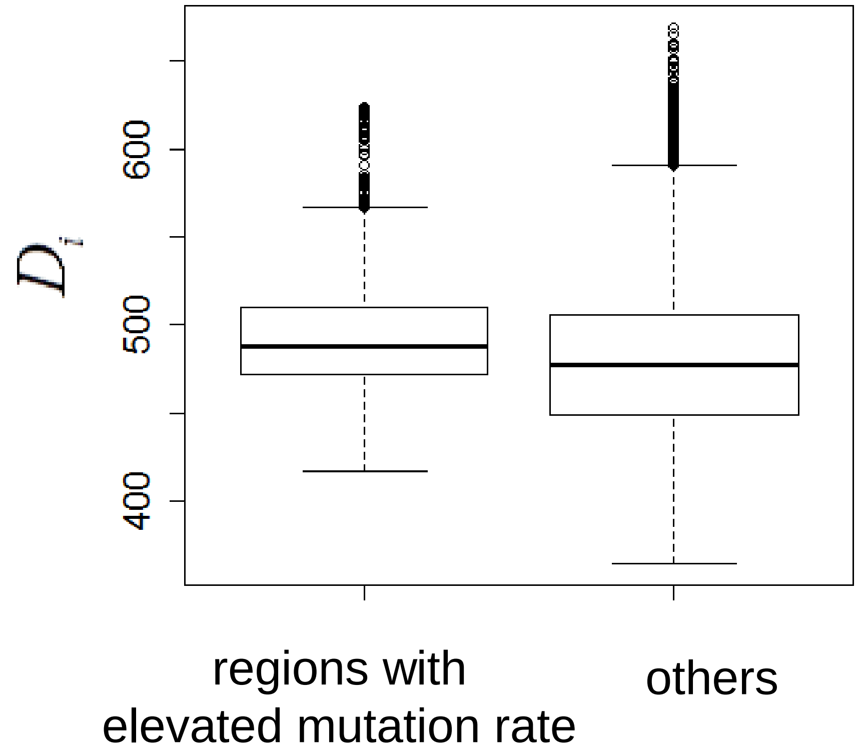
600
500
400
D_i
regions with
elevated mutation rate
others

b.

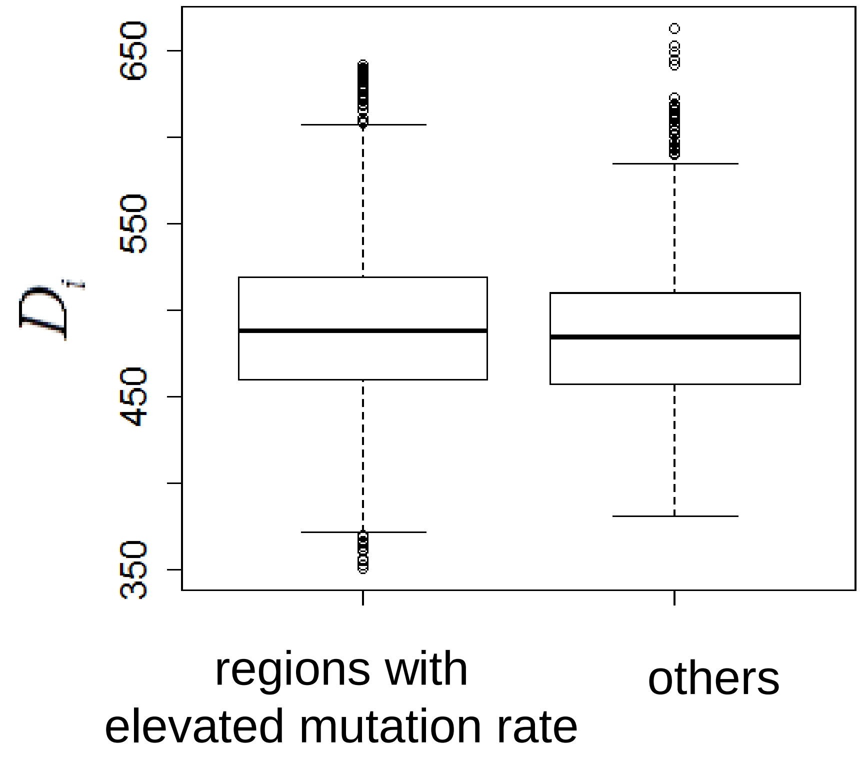
650
550
450
350
D_i
regions with
elevated mutation rate
others

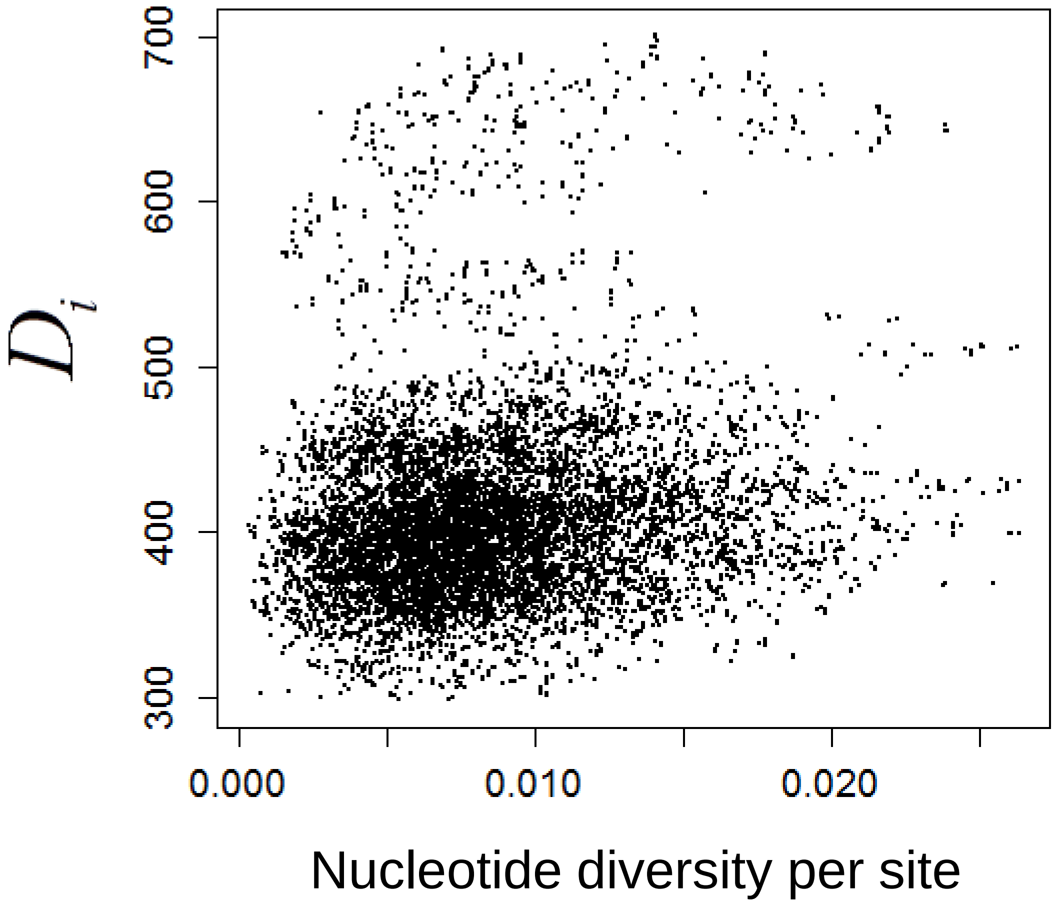

D_i
700
600
500
400
300
0.000
0.010
0.020
Nucleotide diversity per site

a.

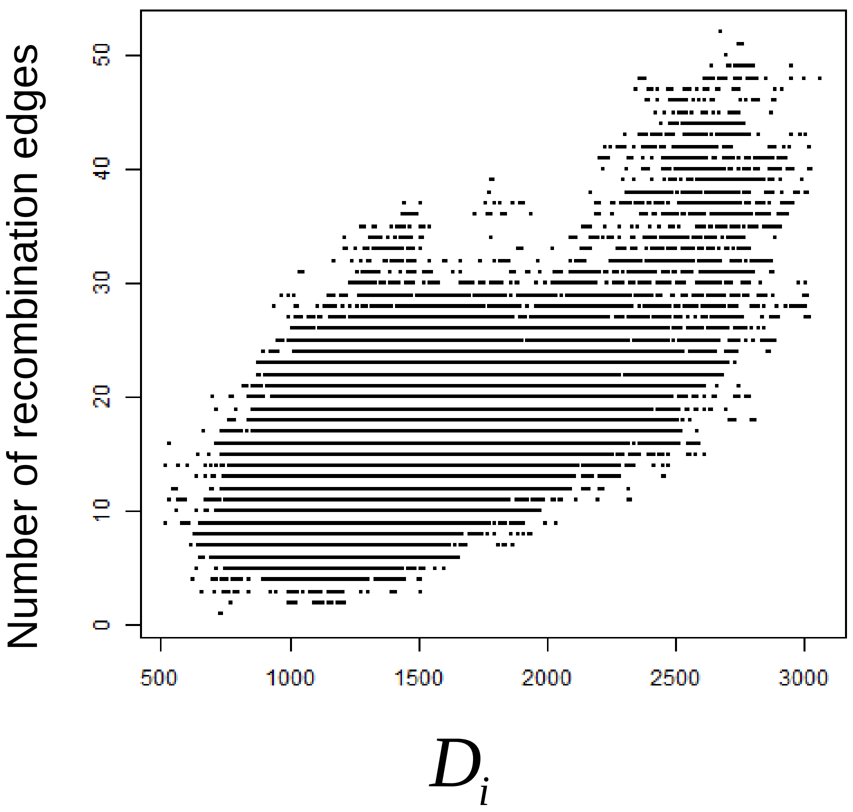
Number of recombination edges
0
10
20
30
40
50
500
1000
1500
2000
2500
3000
D_i

b.

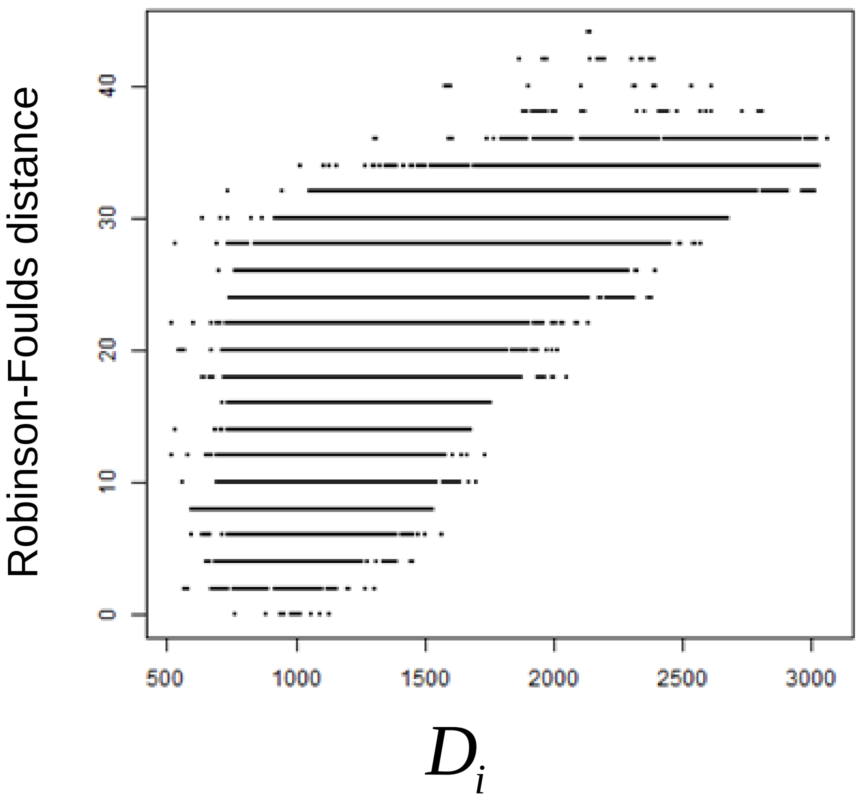
Robinson-Foulds distance
0
10
20
30
40
500
1000
1500
2000
2500
3000
D_i

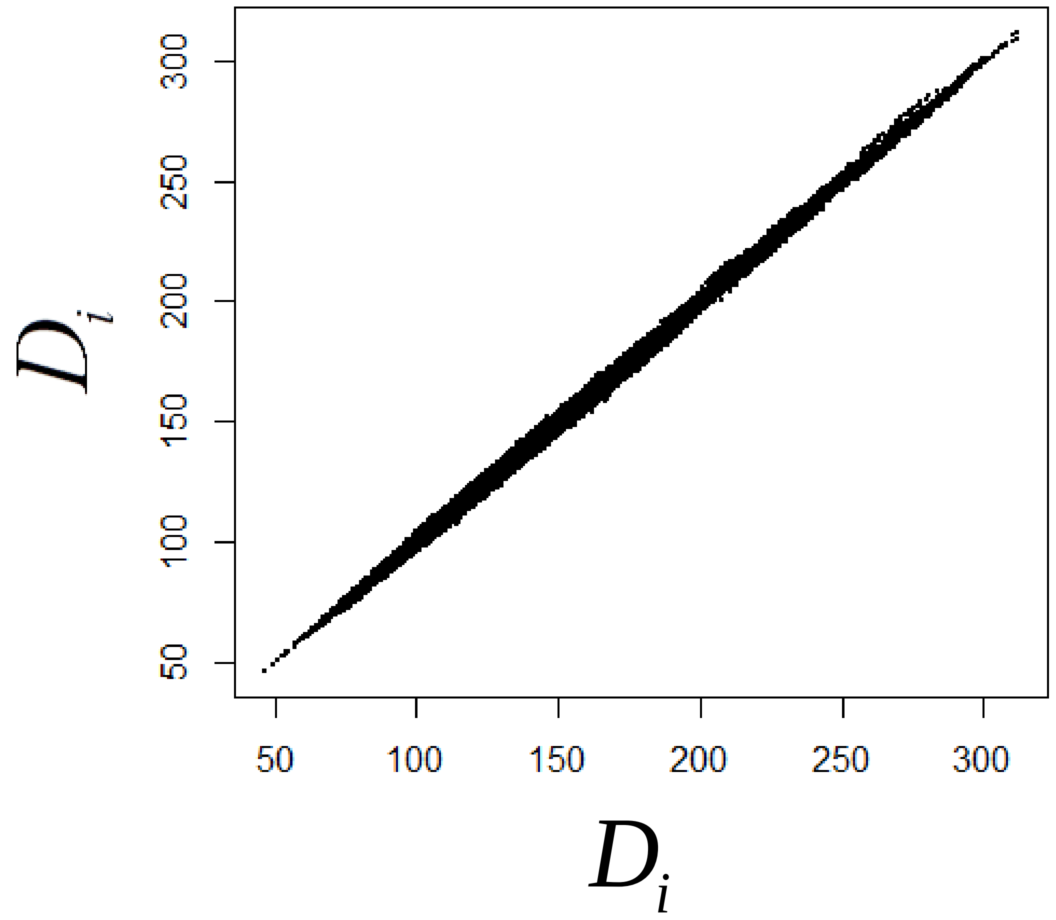

D_i
300
250
200
150
100
50
50
100
150
200
250
300
D_i

a.

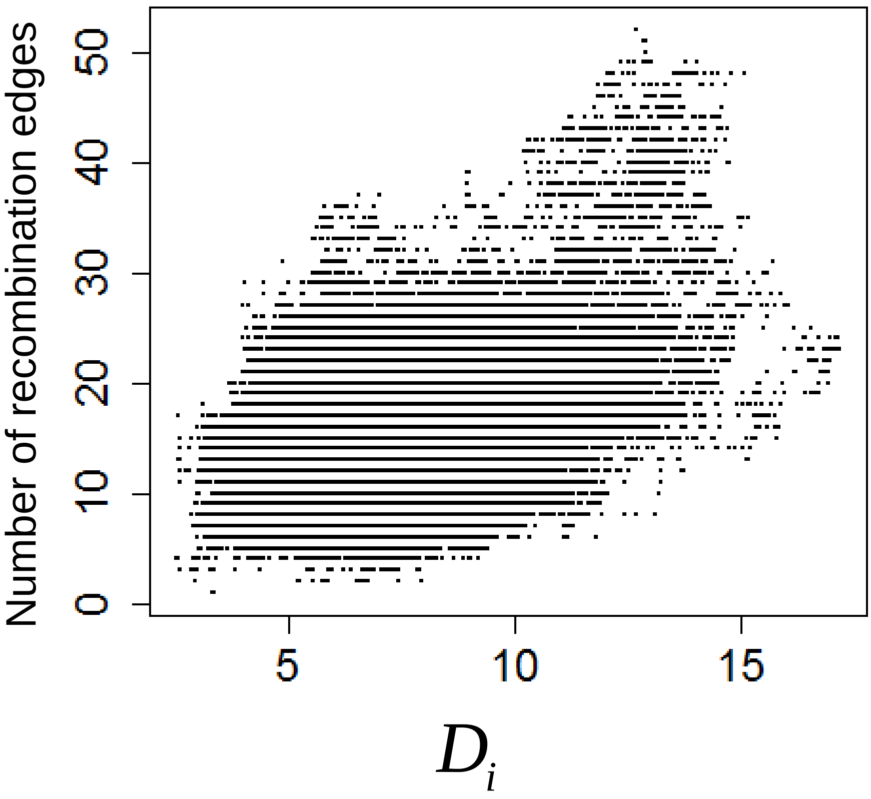
Number of recombination edges
0
10
20
30
40
50
5
10
15
D_i

b.

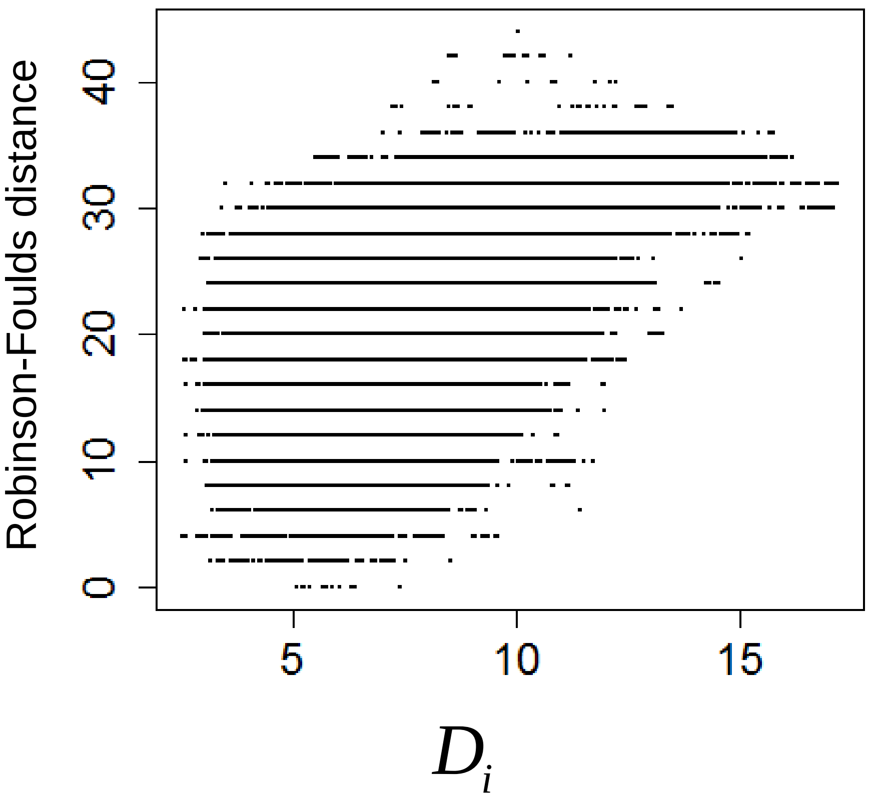
Robinson-Foulds distance
0
10
20
30
40
5
10
15
D_i

Figure S8

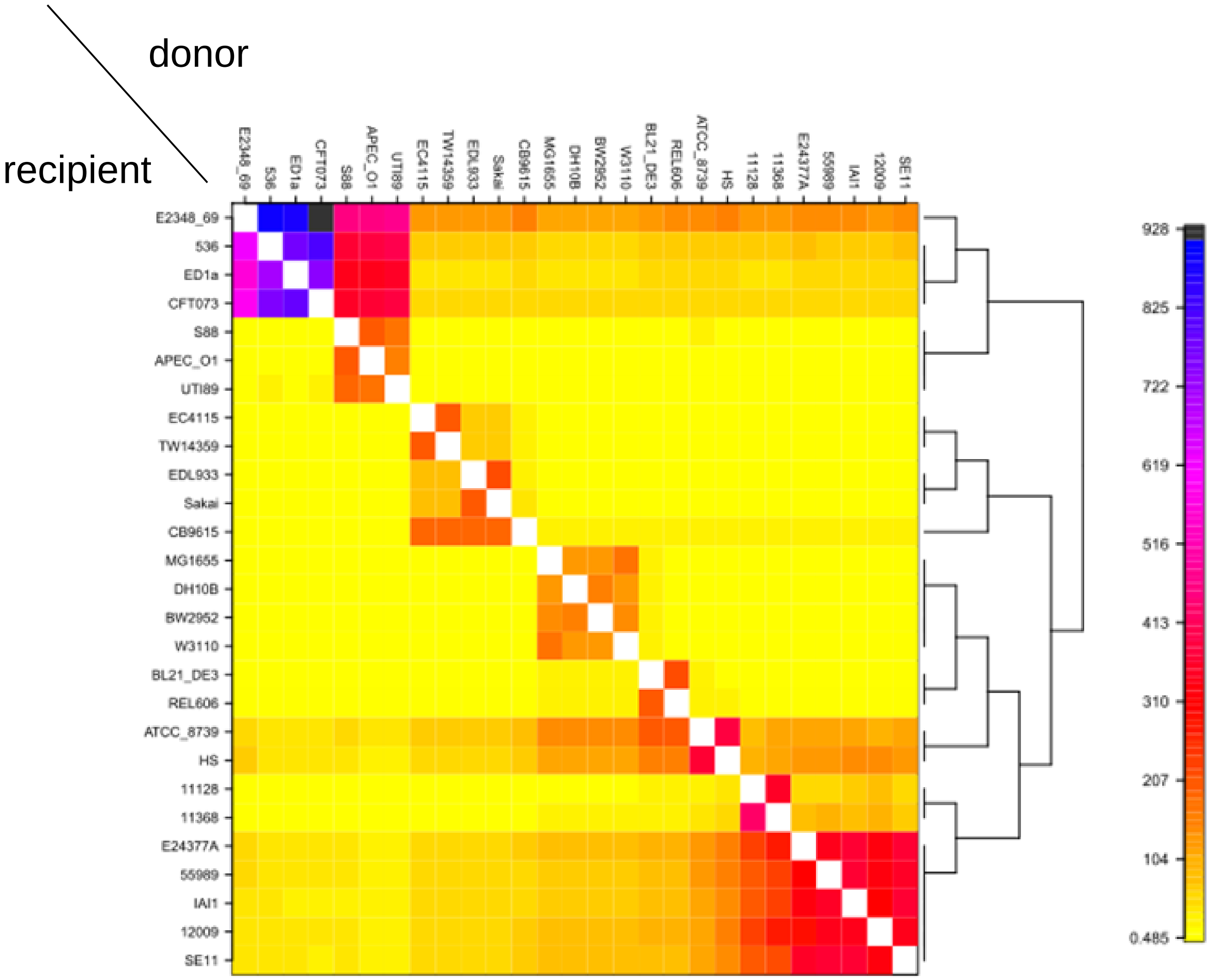
donor
recipient
E2348_69
536
ED1a
CFT073
S88
APEC_O1
UTI89
EC4115
TW14359
EDL933
Sakai
CB9615
MG1655
DH10B
BW2952
W3110
BL21_DE3
REL606
ATCC_8739
HS
11128
11368
E24377A
55989
IAI1
12009
SE11
928
825
722
619
516
413
310
207
104
0.485

a.

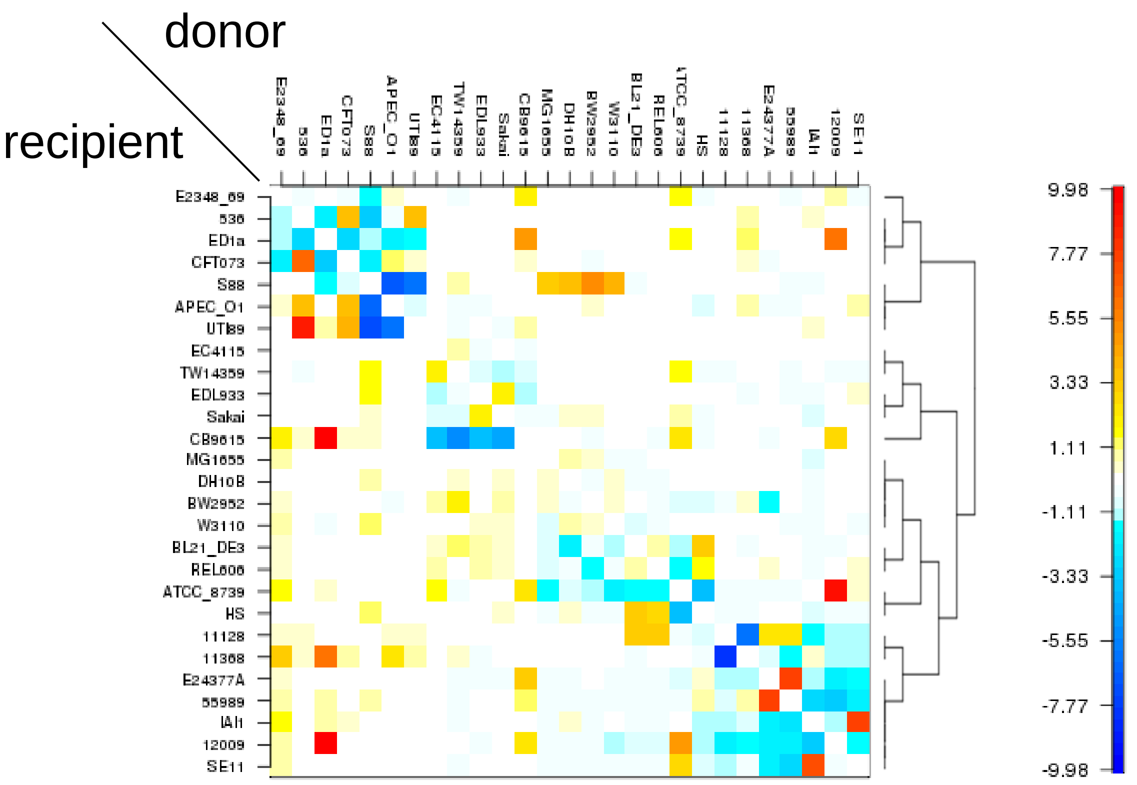
donor
recipient
E2348_69
536
ED1a
CFT073
S88
APEC_O1
UTI89
EC4115
TW14359
EDL933
Sakai
CB9615
MG1655
DH10B
BW2952
W3110
BL21_DE3
REL606
ATCC_8739
HS
11128
11368
E24377A
55989
IAI1
12009
SE11
9.98
7.77
5.55
3.33
1.11
-1.11
-3.33
-5.55
-7.77
-9.98

b.

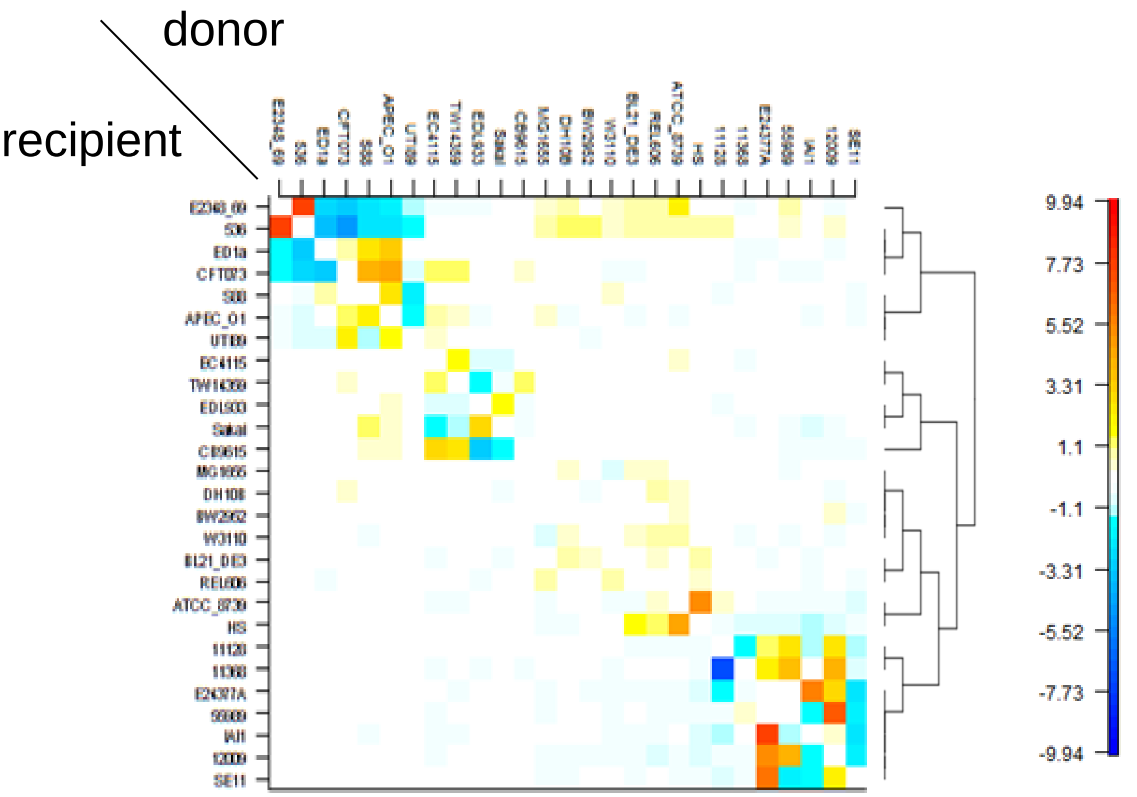
donor
recipient
9.94
7.73
5.52
3.31
1.1
-1.1
-3.31
-5.52
-7.73
-9.94

Figure S10

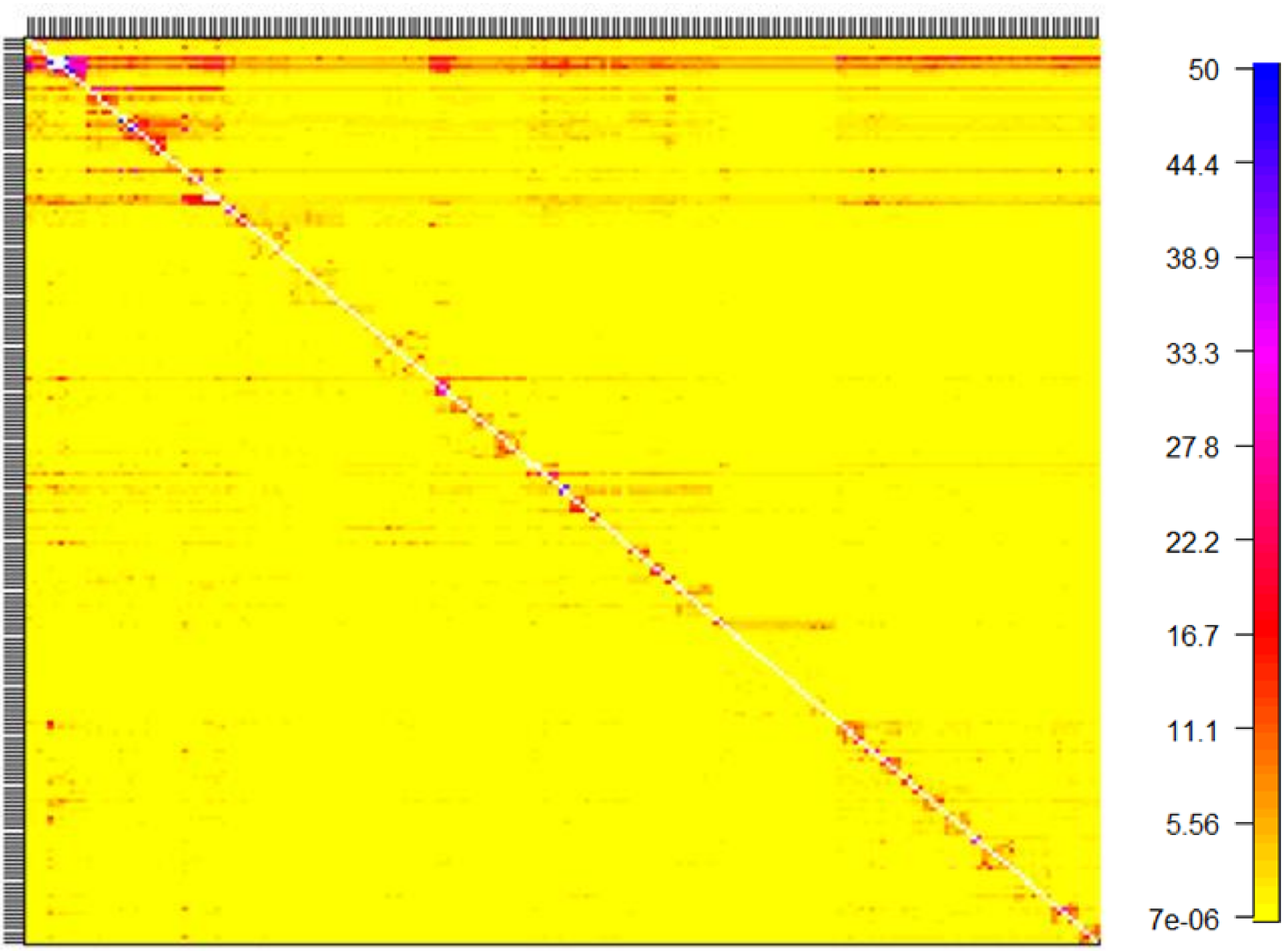

Figure S11

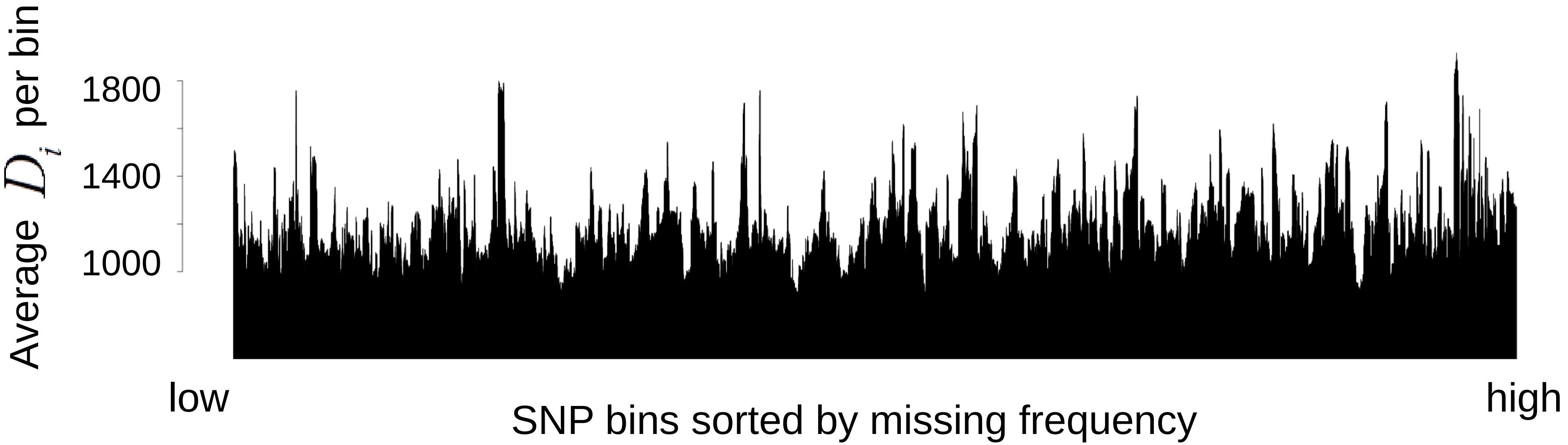

Average D_i per bin
1800
1400
1000
low
high
SNP bins sorted by missing frequency

Figure S12

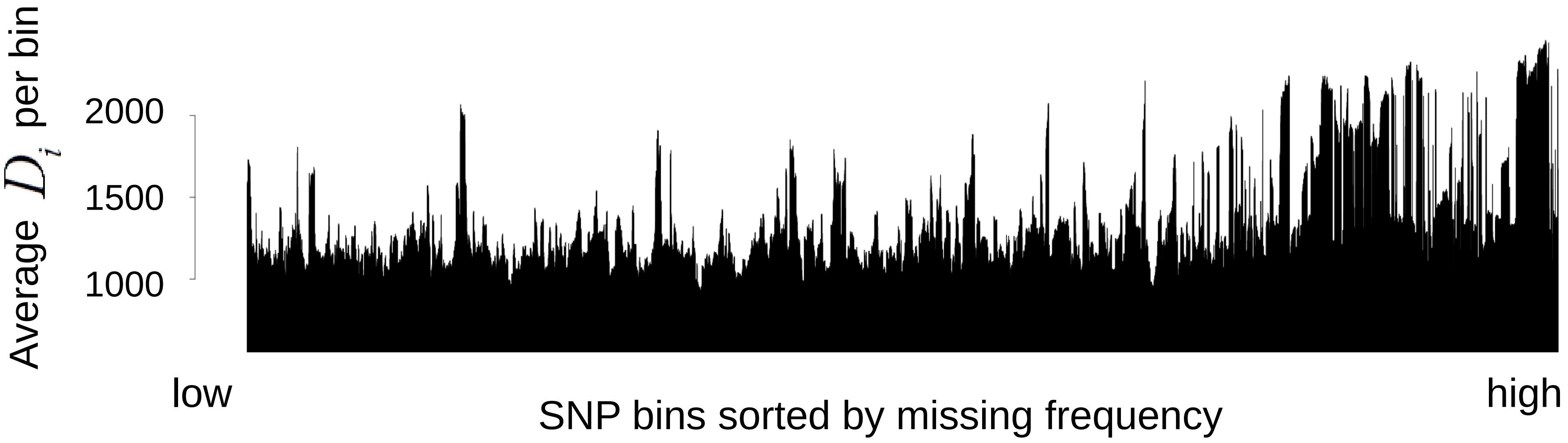
Average D_i per bin
2000
1500
1000
low
high
SNP bins sorted by missing frequency

Figure S13

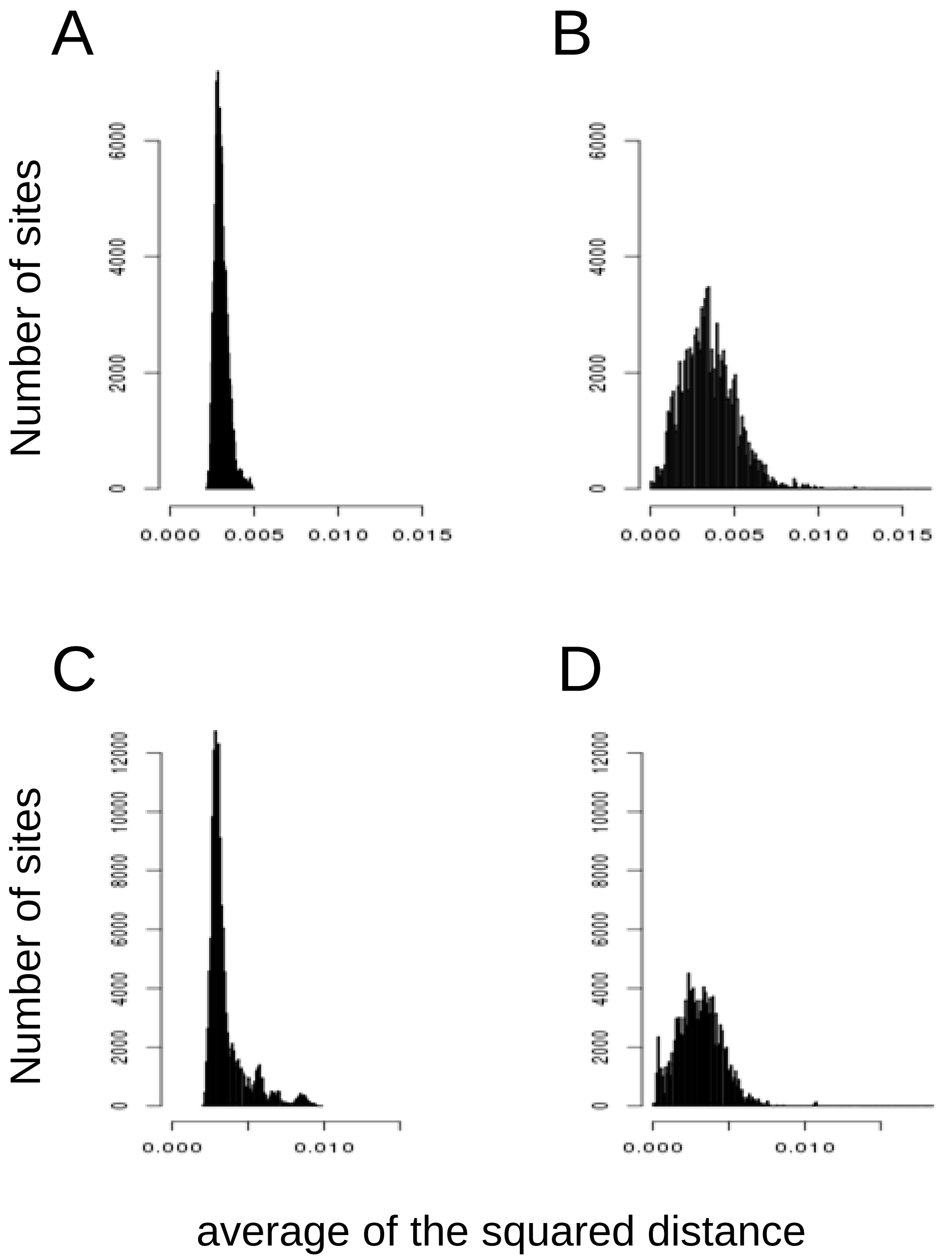
A
B
C
D
Number of sites
Number of sites
0
2000
4000
6000
0
2000
4000
6000
8000
10000
12000
0.000
0.005
0.010
0.015
average of the squared distance

a.

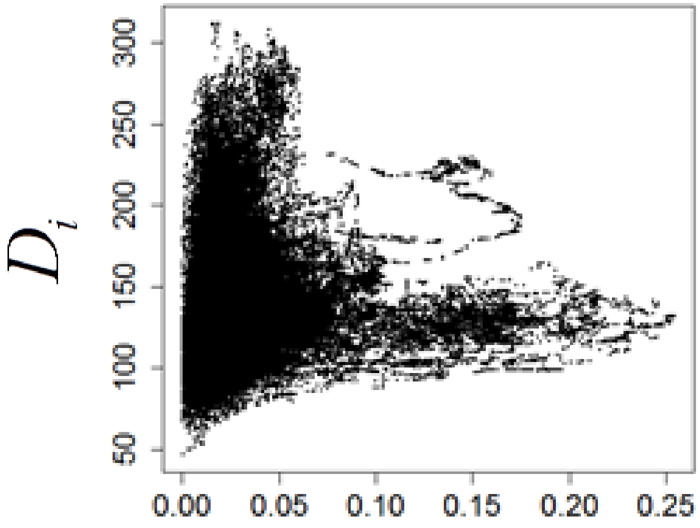

Nucleotide diversity per site

b.

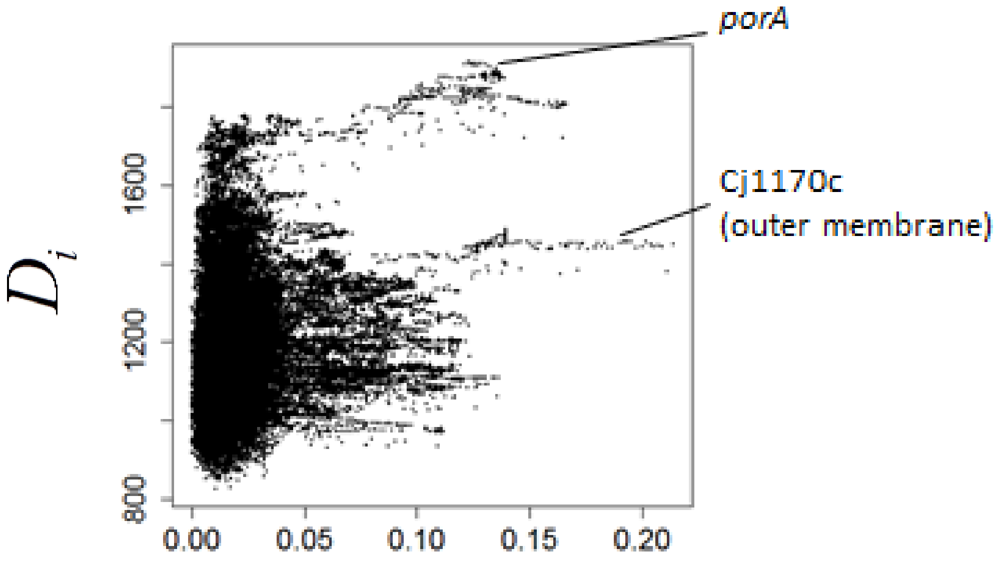

Nucleotide diversity per site

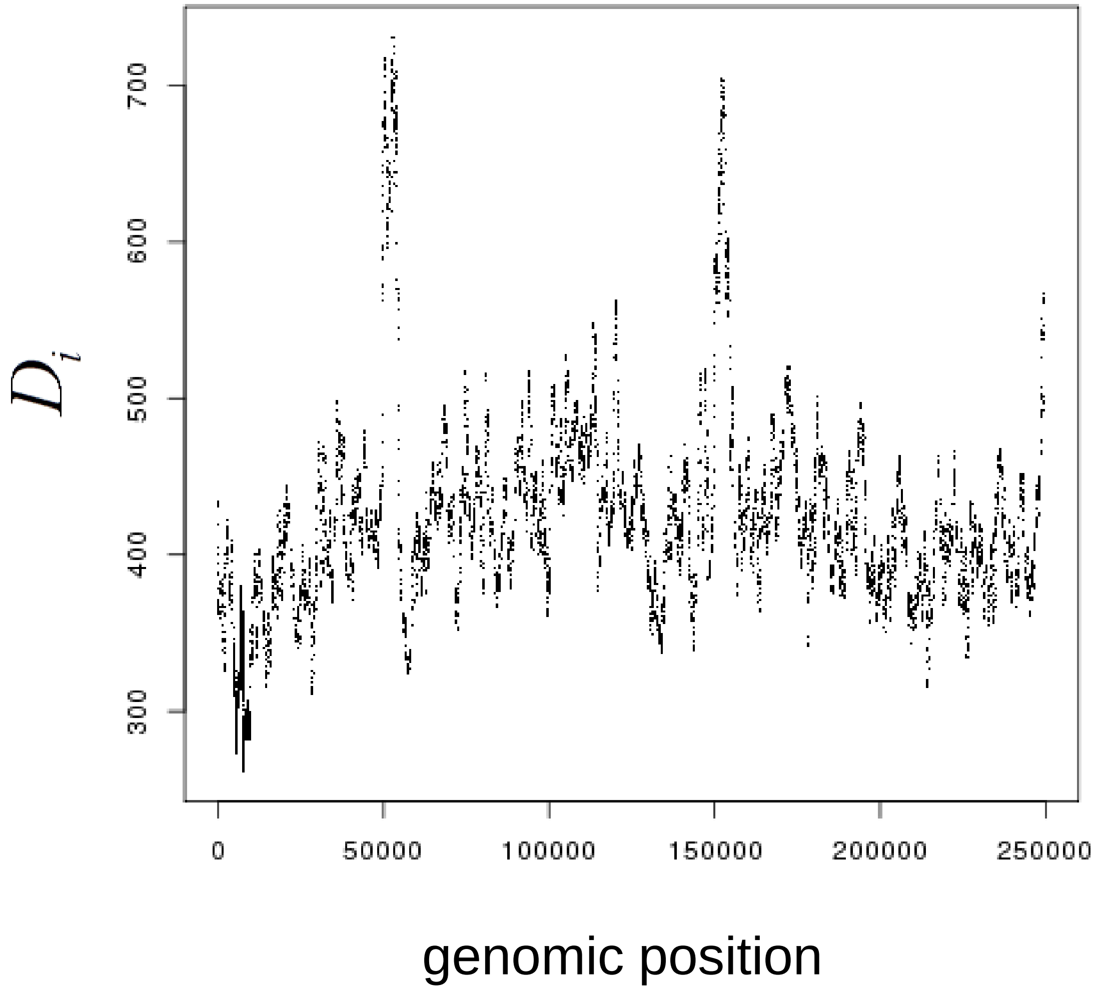
D_i
700
600
500
400
300
0
50000
100000
150000
200000
250000
genomic position

Table S1. Isolate details

Isolate	Alias	Source	Country	Date of isolation	ST[1]	Clonal complex[2]
CAMP45	4	chicken offal or meat	UK	2005	45	ST-45 complex
CAMP61	13	cattle	UK	2006	61	ST-61 complex
CAMP2488	22	chicken	UK	2001	257	ST-257 complex
NCTC 11168 (NC_002163)	29	human	UK		43	ST-21 complex
CampsClin11	32	human unspecified	UK	2005	11	ST-45 complex
CampsClin262	34	human unspecified	UK	2005	262	ST-21 complex
CampsClin266	36	human unspecified	UK	2006	266	ST-21 complex
CampsClin883	37	human unspecified	UK	2006	883	ST-21 complex
chick2219	39	chicken offal or meat	UK	2005	2219	ST-45 complex
chicka21	40	chicken offal or meat	UK	2006	21	ST-21 complex
cow42	42	cattle	UK	2006	42	ST-42 complex
chick594	45	chicken offal or meat	UK	2006	583	ST-45 complex
cow206	48	cattle	UK	2006	206	ST-206 complex
cow38	49	cattle	UK	2006	38	ST-48 complex
cow334	52	cattle	UK	2006	334	ST-45 complex
chick267	54	chicken offal or meat	UK	2005	267	ST-283 complex
CampsClin230	55	human unspecified	UK	2006	230	ST-45 complex
cowa45	56	cattle	UK	2006	45	ST-45 complex
chick2213	57	chicken offal or meat	UK	2005	334	ST-45 complex
cow518	59	cattle	UK	2006	21	ST-21 complex
CampsClin53	60	human unspecified	UK	2005	53	ST-21 complex
cowa21	62	cattle	UK	2006	21	ST-21 complex
chickc21	63	chicken offal or meat	UK	2006	21	ST-21 complex
chick25	64	chicken offal or meat	UK	2006	814	ST-661 complex
chick104	65	chicken offal or meat	UK	2006	104	ST-21 complex
chick353	66	chicken	UK	2009	353	ST-353 complex
chickb354	67	chicken	UK	2009	354	ST-354 complex
chick573	68	chicken	UK	2009	573	ST-573 complex
chick2568	69	chicken	UK	2009	2568	ST-661 complex
chickc45	70	chicken	UK	2009	45	ST-45 complex
chick19	71	chicken	UK	2009	50	ST-21 complex
chick50	72	chicken	UK	2009	50	ST-21 complex
chick53	73	chicken	UK	2009	53	ST-21 complex
chick262	74	chicken	UK	2009	262	ST-21 complex
chick266	75	chicken	UK	2009	266	ST-21 complex
chick1086	77	chicken	UK	2009	50	ST-21 complex
chick1360	78	chicken	UK	2009	50	ST-21 complex
chick11	79	chicken	UK	2009	11	ST-45 complex
chick137	80	chicken	UK	2009	2030	ST-257 complex
chick1003	81	chicken	UK	2009	1003	ST-45 complex
chick2048	82	chicken	UK	2009	45	ST-45 complex
chick2197	83	chicken	UK	2009	354	ST-354 complex
chick2223	84	chicken	UK	2009	45	ST-45 complex
cow3583	85	cattle	UK	2003	3583	ST-42 complex
cow618	86	cattle	UK	2003	61	ST-61 complex
cow273	87	cattle	UK	2003	273	ST-206 complex
cow270	88	cattle	UK	2003	270	ST-403 complex
cowb21	89	cattle	UK	2003	21	ST-21 complex
cowb45	90	cattle	UK	2003	45	ST-45 complex
cowc45	91	cattle	UK	2003	45	ST-45 complex
cowd45	92	cattle	UK	2003	45	ST-45 complex
cow104	94	cattle	UK	2003	104	ST-21 complex
cow3201	97	cattle	UK	2003	19	ST-21 complex
cow3205	99	cattle	UK	2003	206	ST-206 complex
cow137	100	cattle	UK	2003	137	ST-45 complex

cow583	102	cattle	UK	2003	583	ST-45 complex
cow3207	103	cattle	UK	2003	334	ST-45 complex
cow3214	104	cattle	UK	2003	45	ST-45 complex
chick354	105	chicken	UK	2004	257	ST-257 complex
chick51	106	chicken	UK	2005	51	ST-443 complex
chick1079	107	chicken	UK	2004	1079	ST-573 complex
chick574	108	chicken	UK	2004	574	ST-574 complex
chick814	109	chicken	UK	2004	814	ST-661 complex
chickb21	110	chicken	UK	2003	21	ST-21 complex
chickb45	111	chicken	UK	2004	45	ST-45 complex
chickd45	112	chicken	UK	2004	45	ST-45 complex
chick883	113	chicken	UK	2004	883	ST-21 complex
chick230	114	chicken	UK	2004	230	ST-45 complex
OxClina21	117	human unspecified	UK	2003	21	ST-21 complex
OxClina45	119	human unspecified	UK	2003	45	ST-45 complex
Hn129	254	human stool	UK	2003	19	ST-21 complex
Hn30	255	human stool	UK	2003	50	ST-21 complex
Hn39	256	human stool	UK	2003	53	ST-21 complex
Hn4	257	human stool	UK	2003	104	ST-21 complex
Hn123	258	human stool	UK	2003	262	ST-21 complex
Hn4503	259	human stool	UK	2009	19	ST-21 complex
Hn4249	260	human stool	UK	2009	50	ST-21 complex
Hn4075	261	human stool	UK	2009	104	ST-21 complex
Hn3817	262	human stool	UK	2008	262	ST-21 complex
Hn1211	263	human stool	UK	2005	661	ST-661 complex
Hn1550	264	human stool	UK	2006	661	ST-661 complex
13254	265	beef offal or meat	UK	1998	21	ST-21 complex
13255	266	human stool	UK	1991	22	ST-22 complex
13256	267	human stool	UK	1991	42	ST-42 complex
13257	268	human stool	UK	1999	45	ST-45 complex
13258	269	lamb offal or meat	UK	1998	48	ST-48 complex
13259	270	human stool	UK	1991	49	ST-49 complex
13261	272	beef offal or meat	UK	1998	61	ST-61 complex
13262	273	sand (bathing beach)	UK	1994	177	ST-177 complex
13263	274	human stool	UK	1991	206	ST-206 complex
13264	275	human stool	UK	1999	257	ST-257 complex
13265	276	human stool	UK	1991	354	ST-354 complex
13266	277	human stool	UK	1994	362	ST-362 complex
SS_060	278	carcass swab	UK	2008	4468	ST-661 complex
SS_069	281	carcass swab	UK	2008	574	ST-574 complex
SS_077	283	carcass swab	UK	2008	48	ST-48 complex
SS_092	285	carcass swab	UK	2008	353	ST-353 complex
SS_121	286	carcass swab	UK	2008	2030	ST-257 complex
SS_133	288	carcass swab	UK	2008	137	ST-45 complex
SS_153	290	carcass swab	UK	2008	21	ST-21 complex
SS_174	295	carcass swab	UK	2008	3895	ST-353 complex
SS_202	299	carcass swab	UK	2008	257	ST-257 complex
SS_204	300	carcass swab	UK	2008	354	ST-354 complex
SS_208	301	carcass swab	UK	2008	464	ST-464 complex
SS_210	302	carcass swab	UK	2008	1489	ST-354 complex
SS_214	303	carcass swab	UK	2008	48	ST-48 complex
SS_216	304	carcass swab	UK	2008	51	ST-443 complex
SS_223	306	carcass swab	UK	2008	702	ST-702 complex
SS_242	308	carcass swab	UK	2008	354	ST-354 complex
SS_249	309	carcass swab	UK	2008	45	ST-45 complex
SS_256	311	carcass swab	UK	2008	257	ST-257 complex
SS_257	312	carcass swab	UK	2008	583	ST-45 complex
SS_300	315	carcass swab	UK	2008	573	ST-573 complex
SS_307	317	carcass swab	UK	2008	775	ST-52 complex

SS_313	320	carcass swab	UK	2008	19	ST-21 complex
SS_315	321	carcass swab	UK	2008	45	ST-45 complex
SS_320	323	carcass swab	UK	2008	607	ST-607 complex
SS_322	324	carcass swab	UK	2008	45	ST-45 complex
SS_002	325	cattle	UK	2006	19	ST-21 complex
SS_065	330	caecal sample	UK	2008	2030	ST-257 complex
SS_066	331	caecal sample	UK	2008	2135	ST-21 complex
SS_071	334	caecal sample	UK	2009	267	ST-283 complex
SS_079	336	caecal sample	UK	2008	2030	ST-257 complex
SS_081	337	caecal sample	UK	2008	574	ST-574 complex
SS_084	339	caecal sample	UK	2007	775	ST-52 complex
SS_086	340	caecal sample	UK	2009	257	ST-257 complex
SS_091	342	caecal sample	UK	2009	583	ST-45 complex
SS_100	344	caecal sample	UK	2009	3895	ST-353 complex
SS_105	345	caecal sample	UK	2008	3009	ST-433 complex
SS_107	347	caecal sample	UK	2008	48	ST-48 complex
SS_110	348	caecal sample	UK	2009	464	ST-464 complex
SS_113	349	caecal sample	UK	2009	573	ST-573 complex
SS_116	351	caecal sample	UK	2009	2135	ST-21 complex
SS_123	355	caecal sample	UK	2009	257	ST-257 complex
SS_131	357	caecal sample	UK	2008	4460	ST-661 complex
SS_139	359	caecal sample	UK	2008	50	ST-21 complex
SS_141	360	caecal sample	UK	2008	573	ST-573 complex
SS_147	364	caecal sample	UK	2009	863	ST-607 complex
SS_149	365	caecal sample	UK	2007	2314	ST-1034 complex
SS_152	367	caecal sample	UK	2009	2786	ST-661 complex
SS_154	368	caecal sample	UK	2009	48	ST-48 complex
SS_158	369	caecal sample	UK	2007	1408	ST-433 complex
SS_167	370	caecal sample	UK	2009	21	ST-21 complex
SS_169	371	caecal sample	UK	2009	137	ST-45 complex
SS_171	373	caecal sample	UK	2009	2314	ST-1034 complex
SS_172	374	caecal sample	UK	2009	353	ST-353 complex
SS_173	375	caecal sample	UK	2009	574	ST-574 complex
SS_175	376	caecal sample	UK	2008	21	ST-21 complex
SS_177	378	caecal sample	UK	2008	137	ST-45 complex
SS_178	379	caecal sample	UK	2008	21	ST-21 complex
SS_179	380	caecal sample	UK	2008	702	ST-702 complex
SS_185	382	caecal sample	UK	2009	11	ST-45 complex
SS_187	384	caecal sample	UK	2009	50	ST-21 complex
SS_188	385	caecal sample	UK	2009	2030	ST-257 complex
SS_200	387	caecal sample	UK	2008	464	ST-464 complex
SS_206	389	caecal sample	UK	2008	4468	ST-661 complex
SS_212	390	caecal sample	UK	2009	1489	ST-354 complex
SS_218	392	caecal sample	UK	2009	45	ST-45 complex
SS_220	393	caecal sample	UK	2009	257	ST-257 complex
SS_225	394	caecal sample	UK	2007	353	ST-353 complex
SS_226	395	caecal sample	UK	2009	51	ST-443 complex
SS_227	396	caecal sample	UK	2008	1489	ST-354 complex
SS_231	397	caecal sample	UK	2009	4432	ST-661 complex
SS_235	399	caecal sample	UK	2009	2314	ST-1034 complex
SS_243	401	caecal sample	UK	2008	4472	ST-702 complex
SS_252	405	caecal sample	UK	2007	2197	ST-45 complex
SS_254	406	caecal sample	UK	2008	583	ST-45 complex
SS_259	409	caecal sample	UK	2007	354	ST-354 complex
SS_262	411	caecal sample	UK	2008	574	ST-574 complex
SS_287	412	caecal sample	UK	2008	574	ST-574 complex
SS_289	414	caecal sample	UK	2008	51	ST-443 complex
SS_295	416	caecal sample	UK	2008	45	ST-45 complex
SS_296	417	caecal sample	UK	2007	2030	ST-257 complex

SS_297	418	caecal sample	UK	2007	50	ST-21 complex
SS_301	421	caecal sample	UK	2008	19	ST-21 complex
SS_302	422	caecal sample	UK	2008	775	ST-52 complex
SS_303	423	caecal sample	UK	2007	19	ST-21 complex
SS_304	424	caecal sample	UK	2008	257	ST-257 complex
SS_310	427	caecal sample	UK	2007	574	ST-574 complex
SS_312	428	caecal sample	UK	2008	257	ST-257 complex
SS_314	429	caecal sample	UK	2008	2197	ST-45 complex
SS_317	430	caecal sample	UK	2008	2568	ST-661 complex
SS_005	433	chicken	UK	2010	2030	ST-257 complex
SS_006	434	chicken	UK	2011	45	ST-45 complex
SS_025	435	chicken offal or meat	UK	2005	257	ST-257 complex
SS_027	437	chicken offal or meat	UK	2005	257	ST-257 complex
SS_030	438	chicken offal or meat	UK	2005	48	ST-48 complex
SS_032	440	chicken offal or meat	UK	2005	137	ST-45 complex
SS_033	441	chicken offal or meat	UK	2005	257	ST-257 complex
SS_036	443	chicken offal or meat	UK	2005	25	ST-45 complex
SS_037	444	chicken offal or meat	UK	2005	257	ST-257 complex
SS_038	445	chicken offal or meat	UK	2005	257	ST-257 complex
SS_039	446	chicken offal or meat	UK	2005	233	ST-45 complex
SS_040	447	chicken offal or meat	UK	2005	45	ST-45 complex
SS_041	448	chicken offal or meat	UK	2005	661	ST-661 complex
SS_042	449	chicken offal or meat	UK	2005	25	ST-45 complex
SS_044	451	chicken offal or meat	UK	2005	267	ST-283 complex
SS_045	452	chicken offal or meat	UK	2005	257	ST-257 complex
SS_046	453	chicken offal or meat	UK	2005	137	ST-45 complex
SS_053	458	chicken offal or meat	UK	2005	25	ST-45 complex

[1]Sequence type (ST) was derived from the allelic profile of 7 housekeeping genes by multilocus sequence typing (MLST) and confirmed by whole genome sequencing.

[2]Clonal complexes are defined as including any ST that matches a previously defined central genotype (http://pubmlst.org/campylobacter/) at three or more loci.
